# Protein kinase D regulates positive selection of CD4^+^ thymocytes through phosphorylation of SHP-1

**DOI:** 10.1038/ncomms12756

**Published:** 2016-09-27

**Authors:** Eri Ishikawa, Hidetaka Kosako, Tomoharu Yasuda, Masaki Ohmuraya, Kimi Araki, Tomohiro Kurosaki, Takashi Saito, Sho Yamasaki

**Affiliations:** 1 Division of Molecular Immunology, Medical Institute of Bioregulation, Kyushu University, 3-1-1 Maidashi Higashiku, Fukuoka 812-8582, Japan; 2 Division of Cell Signaling, Fujii Memorial Institute of Medical Sciences, Tokushima University, 3-18-15 Kuramoto-cho, Tokushima 770-8503, Japan; 3 Laboratory for Lymphocyte Differentiation, RIKEN Center for Integrative Medical Sciences, 1-7-22 Suehiro-cho, Tsurumi-ku, Yokohama 230-0045, Japan; 4 Institute of Resource Development and Analysis, Kumamoto University, 2-2-1, Honjo, Chuo-ku, Kumamoto 860-0811, Japan; 5 Laboratory for Lymphocyte Differentiation, WPI Immunology Frontier Research Center (iFReC), Osaka University, 3-1 Yamadaoka, Suita 565-0871, Japan; 6 Laboratory for Cell Signaling, RIKEN Center for Integrative Medical Sciences, 1-7-22 Suehiro-cho, Tsurumi-ku, Yokohama 230-0045, Japan; 7 Laboratory for Cell Signaling, WPI Immunology Frontier Research Center (iFReC), Osaka University, 3-1 Yamadaoka, Suita 565-0871, Japan

## Abstract

Thymic selection shapes an appropriate T cell antigen receptor (TCR) repertoire during T cell development. Here, we show that a serine/threonine kinase, protein kinase D (PKD), is crucial for thymocyte positive selection. In T cell-specific PKD-deficient (PKD2/PKD3 double-deficient) mice, the generation of CD4 single positive thymocytes is abrogated. This defect is likely caused by attenuated TCR signalling during positive selection and incomplete CD4 lineage specification in PKD-deficient thymocytes; however, TCR-proximal tyrosine phosphorylation is not affected. PKD is activated in CD4^+^CD8^+^ double positive (DP) thymocytes on stimulation with positively selecting peptides. By phosphoproteomic analysis, we identify SH2-containing protein tyrosine phosphatase-1 (SHP-1) as a direct substrate of PKD. Substitution of wild-type SHP-1 by phosphorylation-defective mutant (SHP-1^S557A^) impairs generation of CD4^+^ thymocytes. These results suggest that the PKD–SHP-1 axis positively regulates TCR signalling to promote CD4^+^ T cell development.

## Abstract (web summary)

The three isoforms of protein kinase D (PKD) have important but often redundant roles in cell signalling. Here the authors show, by generating PKD2/3 double-deficient mice, that PKD is essential for TCR signalling in thymocytes, and identify SHP-1 as a PKD target critical for development of CD4^+^ T cells.

## Graphical abstract

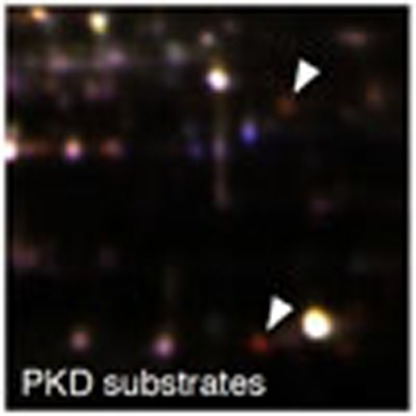

An appropriate αβ T cell receptor (TCR) repertoire is shaped in the thymus through multiple selection steps. In this process, transduction of signals through the TCR in CD4^+^CD8^+^ double positive (DP) thymocytes determines CD4/CD8 lineage specification and generates CD4^+^CD8^−^ and CD4^−^CD8^+^ single positive (SP) thymocytes. At the DP stage, interaction of the TCR with self-peptides on major histocompatibility complex (MHC) molecules generates positive-selecting signals. DP thymocytes that undergo positive selection increase their surface expression of CD5, CD69 and TCR and differentiate into a CD4^+^CD8^int^ transitional stage. At this stage, persistent TCR signalling promotes CD4 lineage specification through a series of transcriptional programs^1^. However, the molecular mechanisms by which the signal duration is translated into specific responses have yet to be fully defined.

It is well established that sequential tyrosine phosphorylation events triggered by protein tyrosine kinases (PTKs), such as Src-, Syk- and Tec-family PTKs, orchestrate TCR signalling during T cell development^2^. Serine/threonine kinases also regulate T cell development by controlling transcriptional and metabolic programs^3^. Until now, loss of several serine/threonine kinases has been reported to result in defective T cell development^4^,^5^,^6^,^7^,^8^,^9^,^10^. However, the crosstalk between serine/threonine kinase and tyrosine phosphorylation cascade is not clearly understood.

PKD, initially called PKCμ, is a serine/threonine kinase family now classified within the CaMK group and separated from the AGC group (named for PKA, PKG and PKC)^11^. The three isoforms, PKD1, PKD2 and PKD3, are encoded by different genes, *prkd1*, *prkd2* and *prkd3*, respectively. All PKD isoforms have a highly conserved amino acid sequence with a diacylglycerol (DAG)-binding domain, pleckstrin homology (PH) domain and kinase domain^11^. Among them, PKD2 is abundantly expressed in T cells^12^,^13^ and is phosphorylated on TCR stimulation^14^. In addition, constitutively active PKD2 drives T cell development^15^. However, thymic selection was not grossly disturbed in PKD2-deficient mice^13^. Until now, no studies have addressed the role of PKD in thymocyte development using mice lacking all PKD isoforms, and the downstream substrates of PKD in thymocytes have not fully been elucidated.

Inhibitory signalling molecules, such as protein tyrosine phosphatases (PTPs), modulate TCR signalling and thus contribute to setting the threshold for thymocyte selection^16^,^17^. In particular, SH2-containing protein tyrosine phosphatase-1 (SHP-1) is reported to regulate thymic selection through a variety of proposed mechanisms^18^,^19^,^20^,^21^. The inhibitory activity of SHP-1 is regulated by binding to the phosphorylated targets via SH2 domains and by phosphorylation of its C-terminal tyrosines (Try^536^ and Tyr^564^)^17^,^22^. However, the role of serine/threonine phosphorylation in the function of SHP-1 remains unclear^23^.

In this study, we established mice lacking all PKD isoforms in T cells. The generation of CD4 SP thymocytes is impaired in these T cell-specific PKD-deficient mice. Using a phosphoproteomic approach, we identify SHP-1 as a direct substrate for PKD. The phosphorylation of SHP-1 by PKD positively regulates TCR signalling. Collectively, we demonstrate that the PKD–SHP-1 axis is required for CD4^+^ T cell development.

## Results

### PKD is phosphorylated in preselection DP thymocytes

**Figure 1:**
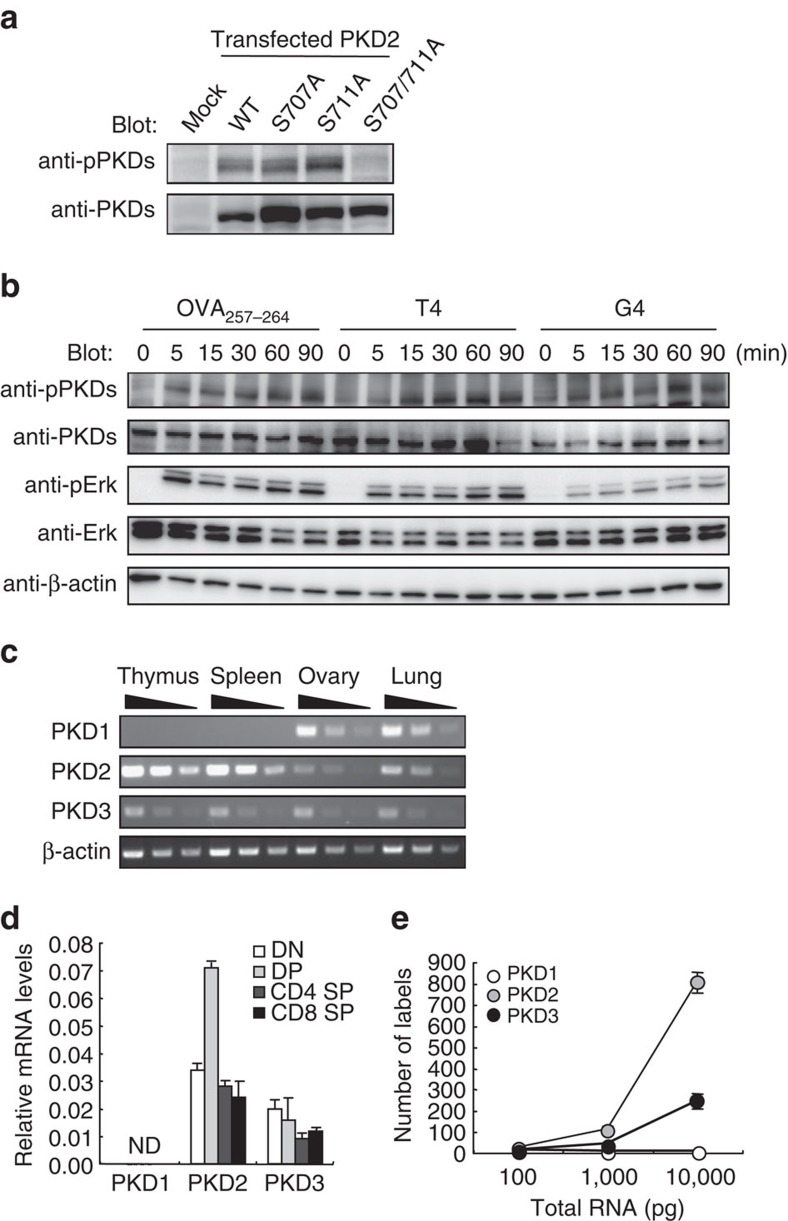
Expression of PKD isoforms in the thymus. (**a**) Characterization of anti-phospho-PKD1/2/3 Abs (anti-pPKDs). Reactivities of anti-pPKDs were evaluated by western blot analysis of lysates from HEK293 cells overexpressing WT PKD2 and mutants. Note that overexpressed PKD2 is constitutively phosphorylated. Anti-PKD1/2/3 (anti-PKDs) blotting was also performed as a control. (**b**) Preselection OT-I DP thymocytes, that are obtained from TAP-deficient mice transferred with OT-I Tg BM cells, were stimulated with a variety of OVA peptides (10 μM) for the indicated times and phosphorylation of PKD and Erk was analysed. PKD, Erk and β-actin were used as loading controls. (**c**) RT-PCR analysis of expression of PKD isoforms in the thymus, spleen, ovary and lung from C57BL/6 mice. mRNA expression of β-actin was analysed as a control. Threefold serial dilutions of cDNAs were used as templates. (**d**) RT-PCR analysis of PKD isoforms expressions in various cell subsets sorted by flow cytometry from WT thymocytes. Results are presented as expression relative to β-actin. ND, not detected. (**e**) mRNA copy number was evaluated by molecular indexing assay. The amount of RNA used in the experiment is indicated on the x axis. Data are representative of two independent experiments (**a**–**c**). Data are presented as mean±s.d. of triplicate assays and representative of two independent experiments (**d**,**e**).

We first investigated whether PKD is activated on TCR engagement in thymocytes by evaluating phosphorylation of its activation loop. To address this, ‘unengaged' DP thymocytes that have never encountered self-peptides were prepared. To obtain preselection thymocytes bearing antigen-specific TCR, bone marrow (BM) cells from OT-I TCR Tg mice were transferred into transporter associated with antigen processing (TAP)-deficient mice, which lack functional MHC class I. The preselection thymocytes were stimulated with OVA peptides that differ in affinity such as OVA_257–264_ (SIINFEKL) (strong agonist), T4 (intermediate agonist) and G4 (weak agonist). To evaluate PKD activation, we established anti-phospho-PKD1/2/3 Abs that recognize phosphorylated activation loops containing a common sequence shared by all PKDs (Fig. 1a). Substantial PKD activation, as assessed by anti-phospho-PKDs Abs, was induced by low-affinity peptides (Fig. 1b), implying a role for PKD in positive selection.

**Figure 2:**
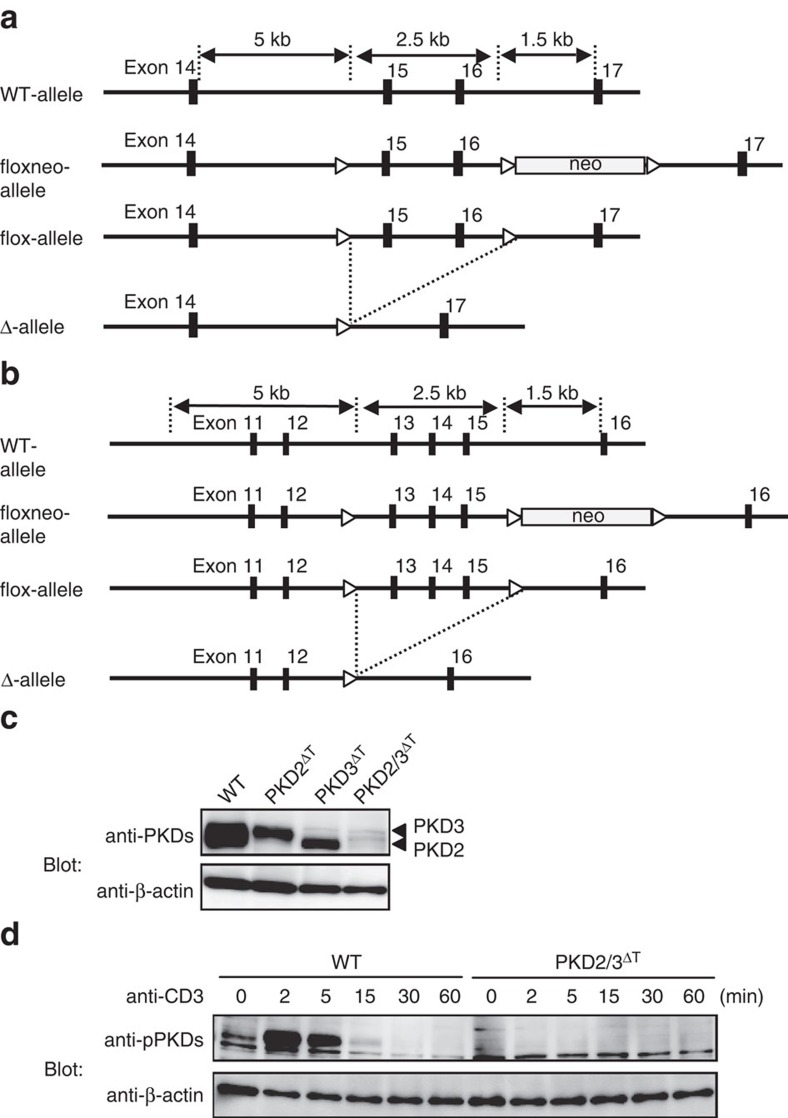
Generation of PKD-deficient mice. (**a**,**b**) Genomic structures and targeting constructs of PKD2 (**a**) and PKD3 (**b**). Exons encoding the DxxxN motif and the phosphorylation sites required for kinase activity are flanked by loxP sites (indicated by open triangles). (**c**) Total thymocytes from WT, PKD2^fl/fl^ × *Lck*-Cre Tg (PKD2^ΔT^), PKD3^fl/fl^ × *Lck*-Cre Tg (PKD3^ΔT^) and PKD2^fl/fl^ × PKD3^fl/fl^ × *Lck*-Cre Tg (PKD2/3^ΔT^) mice were analysed for PKD expression by western blotting using anti-PKDs antibody. β-actin was used as a loading control. (**d**) PKD phosphorylation in thymocytes on TCR stimulation by CD3 cross-linking for 2 min was detected using anti-pPKDs. Data are representative of two independent experiments (**c**,**d**).

We next examined the PKD isoforms expressed in thymocytes. Reverse transcription-PCR (RT-PCR) analyses confirmed that thymocytes express abundant PKD2, less PKD3 and undetectable PKD1 mRNA (Fig. 1c,d), as previously reported^13^. The lack of PKD1 expression in thymocytes was further verified by evaluating mRNA copy number using a molecular indexing assay (Fig. 1e)^24^. To generate T cells lacking all PKD isoforms, we established T cell-specific PKD2/PKD3 double-deficient mice using *Lck*-Cre Tg mice (PKD2/3^ΔT^) (Fig. 2a,b). We confirmed that none of the three PKD isoforms was expressed in PKD2/3^ΔT^ thymocytes using Abs reacting with PKD1, PKD2 and PKD3 (Fig. 2c,d).

### Defective T cell development in PKD2/3^ΔT^ mice

**Figure 3:**
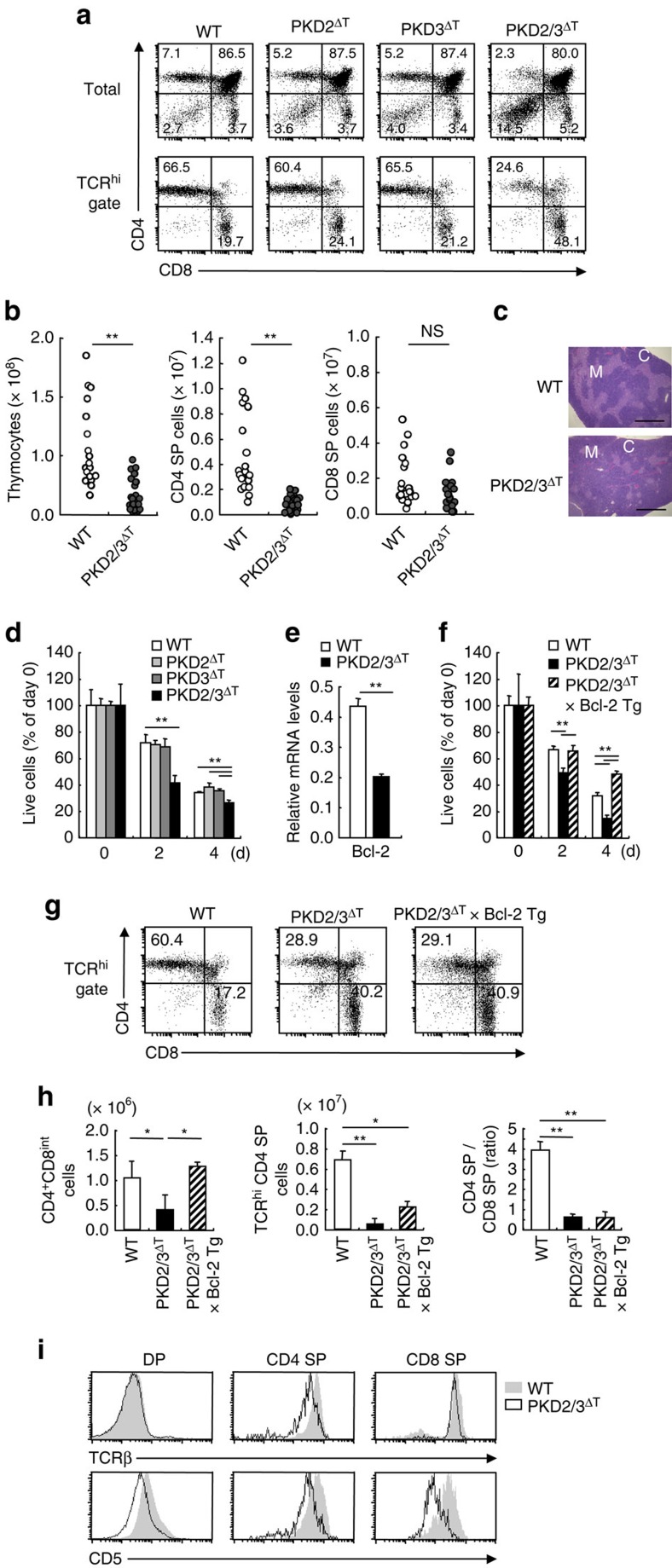
Defective T cell development in the absence of PKD. (**a**) Total thymocytes (upper panels) and TCRβ^hi^ thymocytes (lower panels) from WT, PKD2^fl/fl^ × *Lck*-Cre Tg (PKD2^ΔT^), PKD3^fl/fl^ × *Lck*-Cre Tg (PKD3^ΔT^) and PKD2^fl/fl^ × PKD3^fl/fl^ × *Lck*-Cre Tg (PKD2/3^ΔT^) mice were analysed for CD4 and CD8 expression by flow cytometry. Numbers in quadrants indicate percentages. (**b**) Absolute cell numbers of total thymocytes, CD4 SP and CD8 SP subsets. Each symbol represents an individual mouse. **P<0.01, NS, not significant. (**c**) Hematoxylin and eosin staining of thymus sections. M, medulla; C, cortex. Scale bar, 1 mm. (**d**) *In vitro* survival of thymocytes. Total thymocytes from WT, PKD2^ΔT^, PKD3^ΔT^ and PKD2/3^ΔT^ mice were cultured *in vitro* and the live cell number of CD4 SP cells was analysed by staining with Annexin V and propidium iodide followed by flow cytometry analysis after the indicated numbers of days. **P<0.01. (**e**) Real time PCR analysis of Bcl-2 mRNA expression in CD4^+^CD8^int^ thymocytes. Result is presented as expression relative to β-actin. **P<0.01. (**f**) Analysis of PKD2/3^ΔT^ × Bcl-2 Tg mice. *In vitro* survival of thymocytes from WT, PKD2/3^ΔT^ and PKD2/3^ΔT^ × Bcl-2 Tg mice were analysed as in (**d**). **P<0.01. (**g**) Flow cytometry analysis of CD4 and CD8 expression on TCRβ^+^ thymocytes from WT, PKD2/3^ΔT^ and PKD2/3^ΔT^ × Bcl-2 Tg mice. (**h**) Average cell number of CD4^+^CD8^int^ and CD4 SP thymocytes and the ratio of CD4 SP to CD8 SP thymocytes. *P<0.05, **P<0.01. (**i**) TCRβ and CD5 expression on DP and CD4 and CD8 SP thymocytes from WT (filled histograms) and PKD2/3^ΔT^ mice (open histograms). Data are presented as mean±s.d. of triplicate assays (**d**–**f**) and eight mice (**h**) and are representative of five (**a**,**i**), three (**c**,**f**,**g**), four (**d**) and two (**e**) independent experiments. Unpaired two-tailed Student's *t* test is used to calculate *P* values.

In PKD2/3^ΔT^ mice, the proportion and number of CD4 SP thymocytes were markedly lower compared with wild-type (WT) mice, whereas those from single-deficient (PKD2^ΔT^ or PKD3^ΔT^) mice were not altered (Fig. 3a,b). These trends were more prominent in the TCR^hi^-gated mature CD4 SP population (Fig. 3a, lower panels). Consistent with the reduction of CD4 SP cells, PKD2/3^ΔT^ mice had small and fragmented thymic medulla (Fig. 3c). The above results might be due to a possible survival defect of CD4 SP cells (Fig. 3d; Supplementary Fig. 1), as Bcl-2 expression was reduced by half in PKD2/3^ΔT^ cells (Fig. 3e). However, this possibility seems unlikely as promotion of survival by a Bcl-2 transgene in PKD2/3^ΔT^ mice (Fig. 3f) failed to restore the proportion and number of CD4 SP thymocytes (Fig. 3g,h). Thus, PKD is likely to regulate CD4 SP cell generation beyond any effects on survival. A small number of CD4 SP thymocytes in PKD2/3^ΔT^ mice expressed lower levels of TCR and CD5 on the cell surface (Fig. 3i). This phenomenon was also observed in CD8 SP cells, suggesting that TCR signalling is impaired during positive selection in PKD2/3^ΔT^ mice.

### Role of PKD in positive and negative selection

**Figure 4:**
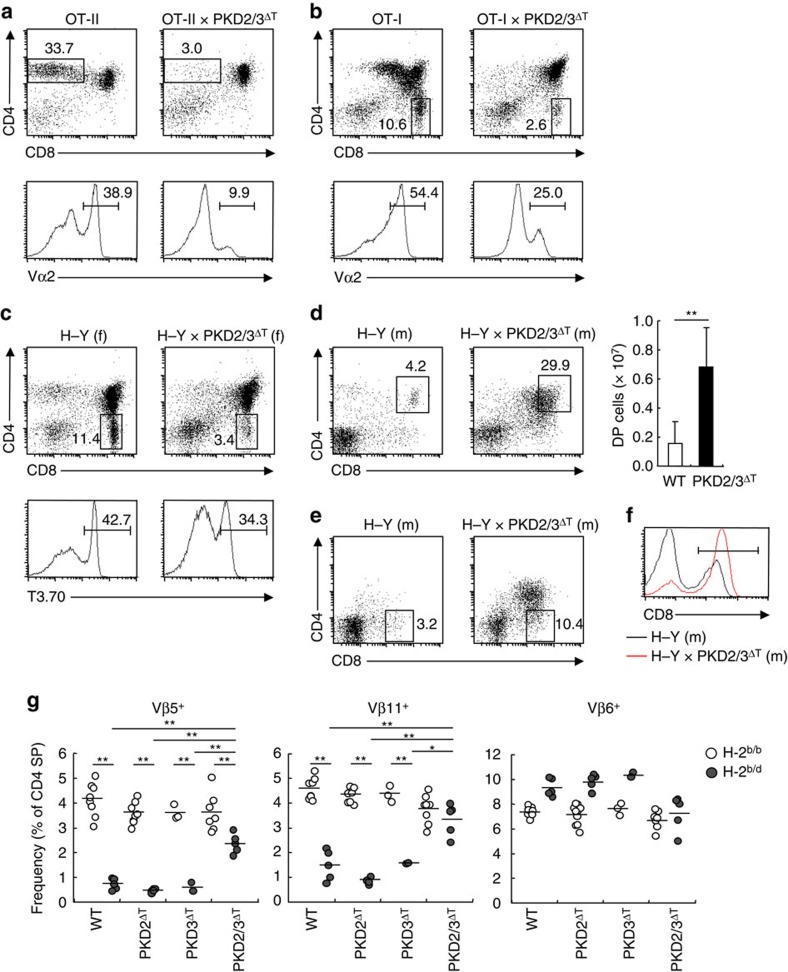
Impaired positive and negative selection in the absence of PKD. (**a**,**b**) Thymocytes from WT and PKD2/3^ΔT^ mice crossed with OT-II TCR Tg (**a**) or OT-I TCR Tg (**b**) were analysed for expression of CD4 and CD8 by flow cytometry. Numbers indicate the percentages of cells in each gate (upper panels). The percentage of Vα2^+^ cells is shown (lower panels). (**c**) Analysis of CD4 and CD8 expression (upper panels) and T3.70 expression (lower panels) of thymocytes from female (f) H–Y TCR Tg mice. (**d**) Analysis of male (m) H–Y TCR Tg mice (left panels). The average number of DP thymocytes is shown (bar graph). **P<0.01. (**e**) T3.70^+^ thymocytes from male H–Y TCR Tg mice were analysed for CD8 SP population. (**f**) T3.70^+^ splenocytes from male H–Y Tg and H–Y Tg × PKD2/3^ΔT^ mice were analysed for CD8 expression. Mean fluorescent intensities (MFIs) of gated population are 208.41 and 337.32, respectively. (**g**) Negative selection induced by endogenous superantigens. Percentage of Vβ5^+^, Vβ11^+^ and Vβ6^+^ cells among CD4 SP thymocytes from B6 (H-2^b^) WT, PKD2^ΔT^, PKD3^ΔT^ and PKD2/3^ΔT^ mice, backcrossed (H-2^b/d^) or not (H-2^b/b^) onto B10.D2 (H-2^d^) mice was analysed by flow cytometry. Each circle represents an individual mouse. Small horizontal lines indicate the mean. *P<0.05, **P<0.01. Data are presented as mean±s.d. of eight mice (**d**, bar graph) and are representative of four independent experiments (**a**–**f**). Unpaired two-tailed Student's *t* test is used to calculate *P* values.

To examine the role of PKD in positive selection, we crossed PKD2/3^ΔT^ mice with MHC class II-restricted OT-II TCR transgenic (Tg) mice. In OT-II × PKD2/3^ΔT^ mice, the percentage of CD4 SP thymocytes was reduced to one-tenth that of PKD-sufficient OT-II mice (Fig. 4a), demonstrating that PKD is critically involved in positive selection for the CD4 lineage. In the MHC class I-restricted OT-I TCR background, the proportion of mature CD8 SP thymocytes in PKD2/3^ΔT^ mice was reduced compared with that in control OT-I Tg mice (Fig. 4b), albeit less severe than in OT-II background. Furthermore, the expression of Tg-TCR was lower in PKD2/3^ΔT^ mice (Fig. 4a,b, lower panels). Analysis of female H–Y TCR Tg mice supported this finding, as CD8 SP development was impaired in H–Y × PKD2/3^ΔT^ mice (Fig. 4c). Thus, positive selection for both the CD4 and CD8 lineages is impaired in PKD2/3^ΔT^ mice, particularly when the αβTCR is fixed by transgenes.

We next investigated the function of PKD in negative selection using two different models. In male H–Y Tg × PKD2/3^ΔT^ mice, the percentage and number of DP thymocytes were significantly higher than in control male H–Y Tg mice, suggesting inefficient negative selection in PKD-deficient thymocytes (Fig. 4d). Strikingly, male H–Y Tg × PKD2/3^ΔT^ mice had CD8 SP thymocytes bearing high levels of the H–Y TCR (Fig. 4e). Furthermore, the peripheral T cells in male H–Y Tg × PKD2/3^ΔT^ mice expressed normal levels of CD8, unlike low-level expression in WT H–Y controls (Fig. 4f). These results suggest that the loss of PKD results in a shift of the normal positive selection window towards high self-reactivity due to impaired TCR signalling in thymocytes.

For the second model, we used a clonal deletion model triggered by endogenous superantigens. Thymocytes bearing Vβ5^+^ and Vβ11^+^ TCR are deleted in the presence of mtv-8 and mtv-9 on I–E (Fig. 4g, H-2^b/d^)^25^. This deletion persisted in PKD single-deficient mice; however, it was significantly blocked in PKD2/3^ΔT^ (H-2^b/d^) mice (Fig. 4g, left panels). Irrelevant Vβ6^+^ cells were not affected by the absence of PKD (Fig. 4g, right panel). Thus, these two models reveal that PKD is involved in negative selection.

Collectively, our results indicate that PKD is critical for positive selection to the CD4 lineage and is also required for optimal positive selection to the CD8 lineage and negative selection.

### Impaired CD4^+^CD8^int^ thymocyte generation by loss of PKD

**Figure 5:**
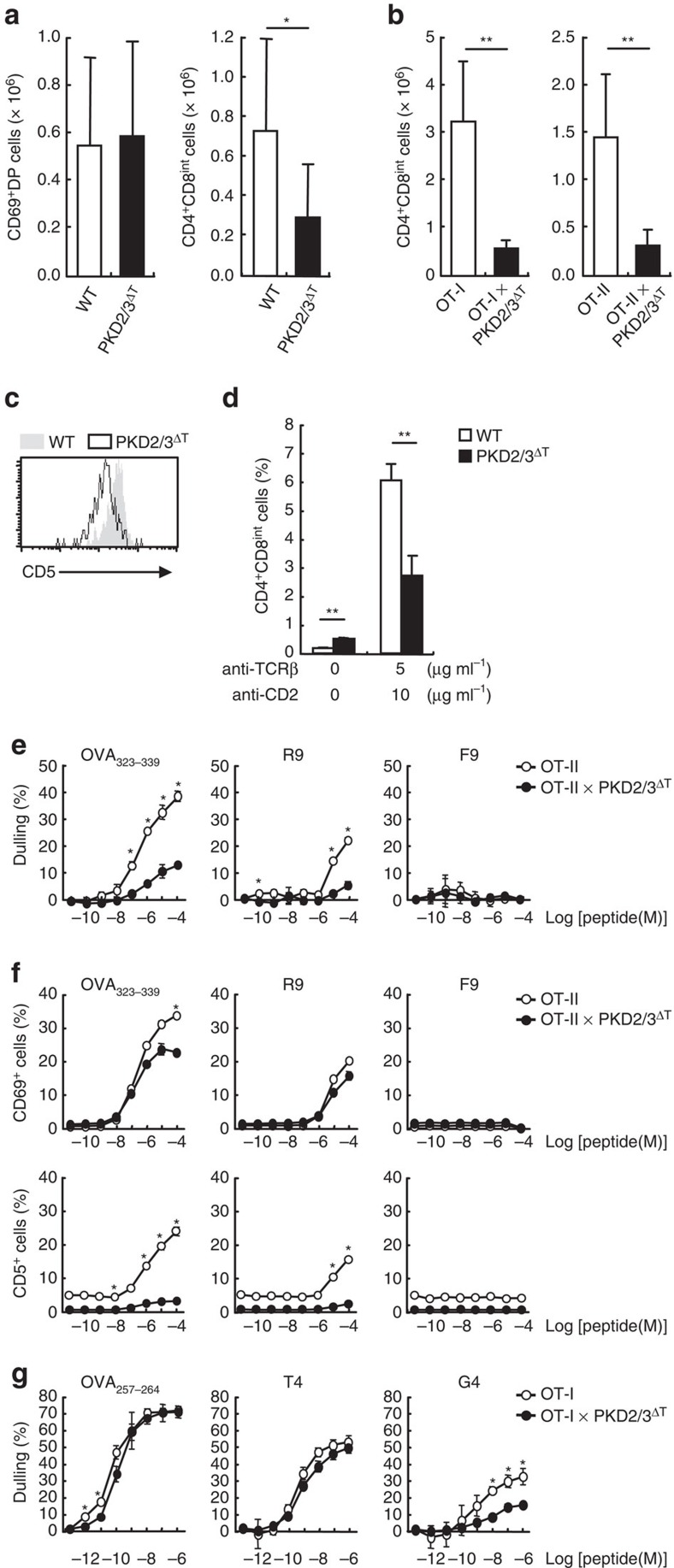
Role of PKD in early differentiation of DP thymocytes. (**a**) Average cell number of CD69^+^DP and CD4^+^CD8^int^ thymocytes from WT and PKD2/3^ΔT^ mice was analysed by flow cytometry. *P<0.05. (**b**) Average cell number of CD4^+^CD8^int^ thymocytes from OT-I or OT-II Tg mice. **P<0.01. (**c**) CD5 expression on TCRβ^hi^CD4^+^CD8^int^ thymocytes from WT and PKD2/3^ΔT^ mice. (**d**) Average percentage of CD4^+^CD8^int^ cells from recovery culture in two-step differentiation assay. **P<0.01. (**e**) Co-receptor downregulation (dulling) on OT-II DP thymocytes on stimulation with OVA peptide variants (OVA_323–339_, R9 and F9) was analysed by flow cytometry. The data are presented as the per cent downregulation, which is the percentage of DP thymocytes with reduced levels of CD4 and CD8 relative to the percentage of DP thymocytes observed in unstimulated condition. *P<0.01. (**f**) Percentage of CD69^+^ cells (upper panels, *P<0.0001) and CD5^+^ cells (lower panels, *P<0.00001) in DP thymocytes on OVA peptides stimulation. (**g**) Co-receptor downregulation (dulling) on preselection OT-I DP thymocytes on stimulation with OVA peptide variants (OVA_257–264_, T4 and G4). The data are presented as in **e**. *P<0.01. Data are presented as mean±s.d. of seven mice (**a**), five mice (**b**) and triplicate assays (**d**) and are representative of five (**c**), three (**d**) or two (**e**–**g**) independent experiments. Unpaired two-tailed Student's *t* test is used to calculate *P* values.

DP thymocytes stimulated with positive-selecting peptides express CD69 and then differentiate into CD4^+^CD8^int^ intermediate cells, which are the precursors to both CD4 and CD8 SP thymocytes^1^. Although the number of CD69^+^ DP cells was not altered in PKD2/3^ΔT^ mice, the CD4^+^CD8^int^ subpopulation was significantly decreased (Fig. 5a). The impaired generation of the CD4^+^CD8^int^ population was also evident in OT-I and OT-II TCR Tg backgrounds (Fig. 5b), indicating that PKD is required for the transition from the DP to the CD4^+^CD8^int^ stage. Consistent with this, the CD4^+^CD8^int^ population in PKD2/3^ΔT^ mice showed lower expression levels of CD5 than in WT mice, suggesting that loss of PKD led to the attenuation of TCR signalling during differentiation of DP to CD4^+^CD8^int^ stage (Fig. 5c).

To confirm this assumption, we conducted two-step differentiation assay *in vitro*. During recovery culture after cross-linking of TCR and co-receptor, DP thymocytes differentiate into CD4^+^CD8^int^ cells (Fig. 5d), as previously reported^26^. However, PKD2/3^ΔT^ DP thymocytes generated a much lower percentage of CD4^+^CD8^int^ cells than WT thymocytes after this treatment (Fig. 5d). Collectively, these results suggest that PKD regulates the TCR signals required for transition of DP to CD4^+^CD8^int^ thymocytes during positive selection.

### PKD mediates signals triggered by weak-affinity peptides

To evaluate the strength of TCR signals, we performed quantitative assays using OVA peptide variants to stimulate thymocytes from TCR Tg mice. Ag peptide-induced co-receptor downregulation is a molecular indicator of the strength of TCR signalling in DP thymocytes^27^. Indeed, stimulation of thymocytes from OT-II TCR Tg mice resulted in co-receptor downregulation in accordance with the peptide affinity as seen using OVA_323–339_ (strong agonist), R9 (weak agonist) and F9 (very weak agonist) (Fig. 5e, open circles)^28^. However, the responses were suppressed in PKD-deficient OT-II thymocytes, even on stimulation with the R9 variant (Fig. 5e, closed circles). Likewise, upregulation of CD69 and CD5 was also reduced in the absence of PKD (Fig. 5f). To further investigate, we stimulated thymocytes bearing OT-I TCR. Again, the co-receptor downregulation faithfully reflected the peptide affinity: namely, OVA_257–264_ is the most potent, followed by T4 and G4 (Fig. 5g, open circles). We found that in the absence of PKD, the response was significantly attenuated when stimulated with a low-affinity variant, G4, which is known as a positive-selecting ligand (Fig. 5g, closed circles)^29^. These findings suggest that PKD is required for TCR signalling triggered by weak-affinity ligands, which is consistent with the above observation that positive selection is severely affected in PKD2/3^ΔT^ mice.

### PKD is involved in CD4 lineage specification

**Figure 6:**
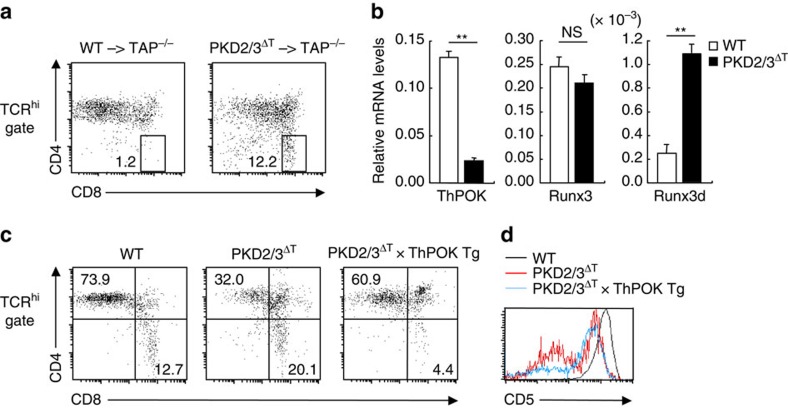
PKD is required for CD4 lineage commitment. (**a**) Analysis of CD8-redirection. BM cells from WT and PKD2/3^ΔT^ mice were transferred into irradiated TAP^−/−^ recipient mice. At 8 weeks after BMT, CD4 and CD8 expression levels on TCRβ^hi^ thymocytes were analysed by flow cytometry. The percentage of gated population is indicated. (**b**) Real time PCR analysis of ThPOK, Runx3 and Runx3d mRNA expressions in CD4^+^CD8^int^ thymocytes. Results are presented as relative expression of β-actin. **P<0.01, NS, not significant. (**c**) Flow cytometry analysis of CD4 and CD8 expression on TCRβ^hi^ thymocytes from WT, PKD2/3^ΔT^ and PKD2/3^ΔT^ × ThPOK Tg mice. (**d**) CD5 expression on CD4 SP thymocytes. Data are presented as mean±s.d. of triplicate assays (**b**) and representative of two (**a**,**b**) or three (**c**,**d**) independent experiments. Unpaired two-tailed Student's *t* test is used to calculate *P* values.

As the generation of CD4 SP cells was more severely affected than CD8 SP cells in PKD2/3^ΔT^ mice (Fig. 3a,b), we next investigated whether PKD influences CD4 lineage commitment. If PKD2/3^ΔT^ thymocytes have an intrinsic defect in commitment to the CD4 lineage, they should give rise to CD8 SP cells even in the absence of MHC class I (in an MHC class II-dependent manner), which is called ‘CD8-redirection' (refs 30, 31). To address this possibility, we transferred BM cells from WT or PKD2/3^ΔT^ mice to TAP-deficient mice. PKD-deficient BM cells, but not WT BM cells, gave rise to mature CD8 SP thymocytes even in TAP-deficient hosts lacking functional MHC class I (Fig. 6a), suggesting that PKD2/3^ΔT^ cells are defective in lineage commitment to CD4 SP thymocytes.

In fact, expression of ThPOK, a critical regulator of CD4 lineage commitment^32^, was decreased in PKD2/3^ΔT^ CD4^+^CD8^int^ cells, whereas Runx3, a transcription factor essential for CD8 lineage specification, was not decreased (Fig. 6b). Forced expression of ThPOK restored the percentage of CD4 SP thymocytes to nearly WT levels (Fig. 6c). However, the expression of the CD5 maturation marker in CD4^+^CD8^int^ cells remained low even in the presence of ThPOK transgene (Fig. 6d). Thus, these results suggest that PKD regulates the development of CD4 SP cells through at least two mechanisms: (i) promotion of TCR signals in the DP stage that trigger transition to the CD4^+^CD8^int^ stage; and (ii) specification of CD4 lineage development via a ThPOK-dependent process.

### SHP-1 is a PKD substrate in thymocytes

**Figure 7:**
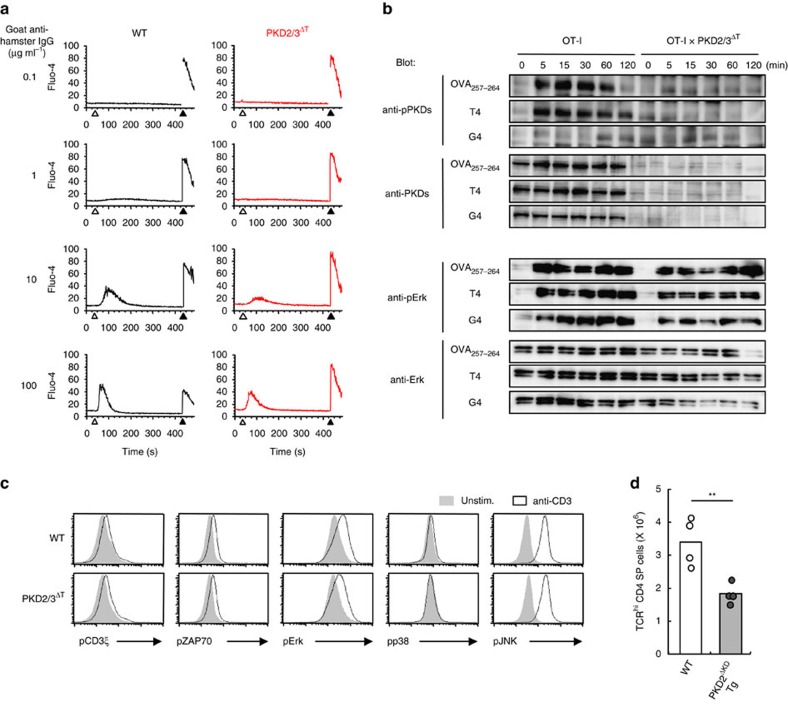
TCR downstream signalling in PKD-deficient thymocytes. (**a**) Ca^2+^ influx in freshly isolated DP thymocytes from WT and PKD2/3^ΔT^ mice after TCR stimulation by CD3 cross-linking using indicated concentration of goat anti-hamster IgG (open arrowhead) followed by the addition of ionomycin (closed arrowhead) was assessed by flow cytometry. (**b**) OT-I preselection DP thymocytes were stimulated with OVA peptide variants for the indicated times and phosphorylation of PKD and Erk was analysed by immunoblotting. PKD and Erk were used as a loading control. (**c**) Phosphorylation of CD3ζ, ZAP-70, Erk, p38 and JNK in unstimulated (filled histograms) and anti-CD3-stimulated DP thymocytes (open histograms) was analysed by intracellular staining. (**d**) Average cell number of TCRβ^hi^ CD4 SP thymocytes from WT and truncated PKD2 Tg mice lacking the kinase domain (PKD2^ΔKD^ Tg) was analysed by flow cytometry. Each circle represents an individual mouse. **P<0.01. Data are representative of three independent experiments (**a**–**c**). Data are presented as mean±s.d. of four mice (**d**, bar graph). Unpaired two-tailed Student's *t* test is used to calculate *P* values.

The above results demonstrate that PKD promotes TCR signalling during thymic selection. However, proximal signalling events including Ca^2+^ influx and Erk activation following TCR ligation were comparable between WT and PKD2/3^ΔT^ thymocytes (Fig. 7a,b). Furthermore, in DP cells, TCR-induced tyrosine phosphorylation of proximal signalling molecules, such as CD3ζ and ZAP-70, as well as MAPKs, such as Erk, p38 and JNK, was unaffected by the loss of PKD (Fig. 7c).

On the other hand, expression of a truncated PKD2 transgene lacking the kinase domain inhibited the generation of CD4 SP thymocytes (Fig. 7d), suggesting that the PKD kinase activity is required for thymocyte development.

**Figure 8:**
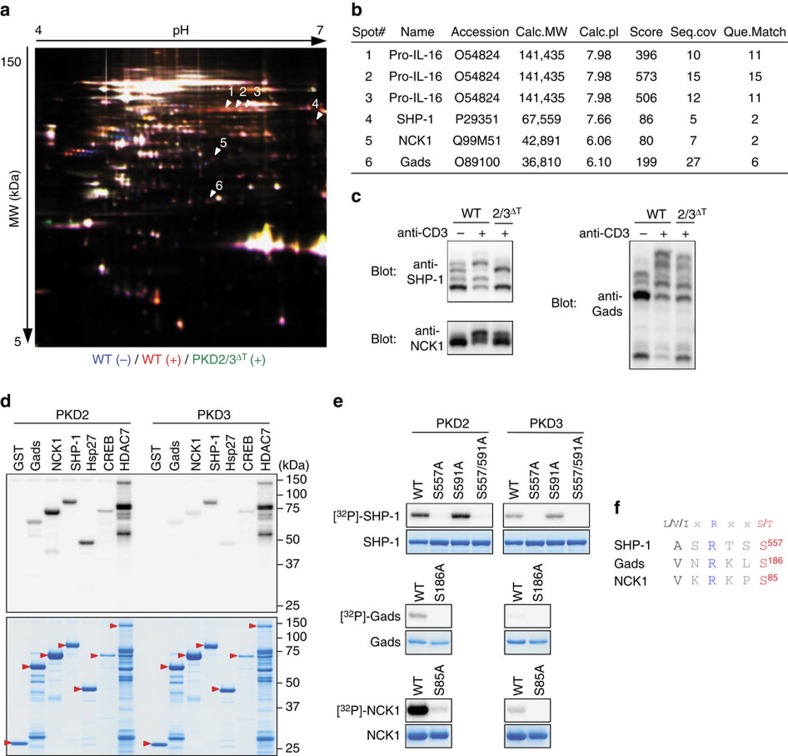
Identification of PKD2 and PKD3 substrates. (**a**) A gel image of 2D-DIGE of phosphoproteins from unstimulated WT thymocytes (Cy2-labelled, blue), WT thymocytes stimulated by CD3 cross-linking (Cy3-labelled, red) and stimulated PKD2/3^ΔT^ thymocytes (Cy5-labelled, green). Red spots are indicated by arrowheads as spot #1 to #6. (**b**) The summary of the protein identity, accession number, calculated molecular weight, calculated pI, Mascot score, sequence coverage and query match for each of six spots in **a**. Spots #1, #2 and #3, which have the same molecular weight with different pI's were identified as pro-IL-16. (**c**) Phos-tag immunoblot analysis of cell lysates from unstimulated- and TCR-stimulated-WT thymocytes for 2 min and stimulated PKD2/3^ΔT^ thymocytes using anti-SHP-1, anti-NCK1 and anti-Gads. (**d**) *In vitro* kinase assays were performed in the presence of [γ-^32^P]ATP with active, recombinant PKD2 or PKD3 and GST-fused substrates as indicated (upper panel). CBB staining shows that equivalent amounts of proteins were used for the assays (lower panel). Red arrowheads indicate GST-fused substrates. (**e**) Identification of phosphorylation sites. *In vitro* kinase assays were performed with PKD2 or PKD3 and, as substrates, WT or mutant SHP-1, Gads and NCK1. Mutant substrates carry serine to alanine substitution at the indicated amino acid positions. CBB staining is shown as a loading control. (**f**) Comparison of the PKD consensus phosphorylation motif and phosphorylation sites identified in **e**. Data are representative of two independent experiments (**a**–**e**).

Hence, to clarify downstream signalling events, we performed an unbiased proteomic search for novel PKD substrates in thymocytes. To identify proteins that exhibit TCR-induced phosphorylation in WT but not PKD-deficient thymocytes, phosphoproteins from unstimulated WT, stimulated WT and stimulated PKD2/3^ΔT^ thymocytes were labelled with Cy2 (blue), Cy3 (red) and Cy5 (green) fluorescent markers, respectively, and analysed by two-dimensional fluorescence difference gel electrophoresis (2D-DIGE)^33^. We detected six ‘red' spots representing proteins that were phosphorylated in a stimulation- and PKD-dependent manner (Fig. 8a). These spots were identified to be pro-IL-16, SHP-1, NCK1 and Gads by mass spectrometry (MS) (Fig. 8a,b; Supplementary Data 1). SHP-1, NCK1 and Gads are reported to be involved in T cell development^17^,^34^,^35^. Phosphate-affinity SDS–PAGE (Phos-tag) western blotting^33^ showed that the migration of SHP-1, NCK1 and Gads was retarded (indicating phosphorylation) in stimulated-WT thymocytes (Fig. 8c; Supplementary Fig. 2). In PKD2/3^ΔT^ cells, the levels of phosphorylation of these proteins were reduced (Fig. 8c). *In vitro* kinase assays with recombinant PKD2 and PKD3 revealed that SHP-1, Gads and NCK1 are direct substrates for PKD2/3, and were phosphorylated at an efficiency similar to substrates previously identified in other tissues (Fig. 8d)^36^. We next used MS and site-directed mutagenesis, and identified the sites phosphorylated by PKD as SHP-1 Ser^557^, Gads Ser^186^ and NCK1 Ser^85^ (Fig. 8e, Supplementary Fig. 3 and Supplementary Data 2). Notably, these sites are similar in sequence to the PKD consensus phosphorylation motif, L/V/IxRxxpS/T (Fig. 8f)^37^.

**Figure 9:**
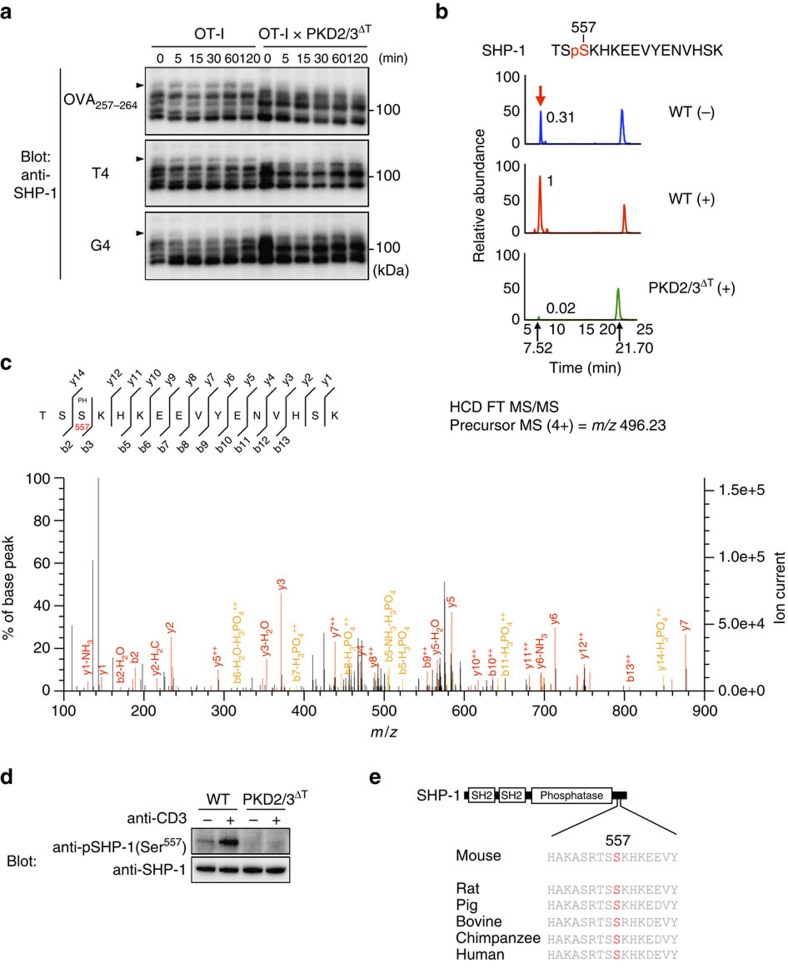
SHP-1 is phosphorylated by PKD2 and PKD3 at Ser^557^ in thymocytes. (**a**) Phosphorylation of SHP-1 on peptide stimulation. OT-I DP thymocytes were stimulated with the designated OVA peptides for the indicated times. Cell lysates were subjected to Phos-tag immunoblot analysis using anti-SHP-1. Arrowheads indicate phosphorylated SHP-1 that showed stimulation-dependent mobility shift. (**b**) The extracted ion chromatogram of *m/z* 496.23096 corresponds to the quadruply charged SHP-1 phosphopeptide (TSpS^557^KHKEEVYENVHSK), which was increased by TCR stimulation in tryptic phosphopeptides from WT thymocytes and was almost undetectable in those from stimulated PKD2/3^ΔT^ thymocytes. The relative values of the peak area are indicated. Note that the hextuply charged ion peak at a retention time of 21.70 min was unrelated to this SHP-1 phosphopeptide. (**c**) Phosphorylation of Ser^557^ was demonstrated by the MS/MS spectrum of the *m*/*z* 496.23 ion at a retention time of 7.52 min in tryptic phosphopeptides from stimulated-WT thymocytes. (**d**) Phosphorylation of Ser^557^ of SHP-1 was analysed by immunoblotting. Total thymocytes were stimulated by CD3 cross-linking for 2 min and analysed by anti-phospho-SHP-1 (Ser^557^) Abs. Total SHP-1 was blotted as a control. (**e**) Schematic structure of SHP-1 and sequence alignment of the C-terminal region of SHP-1 that contains Ser residue corresponding to Ser^557^ of murine SHP-1 (shown in red). Data are representative of two independent experiments (**a**–**d**).

These substrates were also phosphorylated in OT-I thymocytes on stimulation with various OVA peptides as assessed by Phos-tag western blotting. Among these substrates, the phosphorylation of SHP-1 was largely dependent on PKD2/3 (Fig. 9a). In order to further examine SHP-1 phosphorylation *in vivo*, tryptic phosphopeptides were enriched using TiO_2_ from each thymocyte. The liquid chromatography (LC)–MS data revealed that a phosphorylated peptide corresponding to amino acid residues 555–570 was increased by TCR stimulation in a PKD2/3-dependent manner (Fig. 9b, Supplementary Fig. 4 and Supplementary Data 3). Tandem mass spectrometry (MS/MS) analysis confirmed phosphorylation at Ser^557^ of endogenous SHP-1 derived from stimulated-WT thymocytes (Fig. 9c). Finally, we developed an antibody that recognizes the phosphorylated Ser^557^ in SHP-1. TCR stimulation increased the levels of phospho-Ser^557^ SHP-1 in thymocytes from WT but not PKD2/3^ΔT^ mice (Fig. 9d). These results indicate that PKD mediates TCR-induced phosphorylation of Ser^557^ in SHP-1. Importantly, the Ser^557^ amino acid is highly conserved across mammalian species (Fig. 9e).

### Mutant SHP-1 (SHP-1^S557A^) suppresses thymocyte development

**Figure 10:**
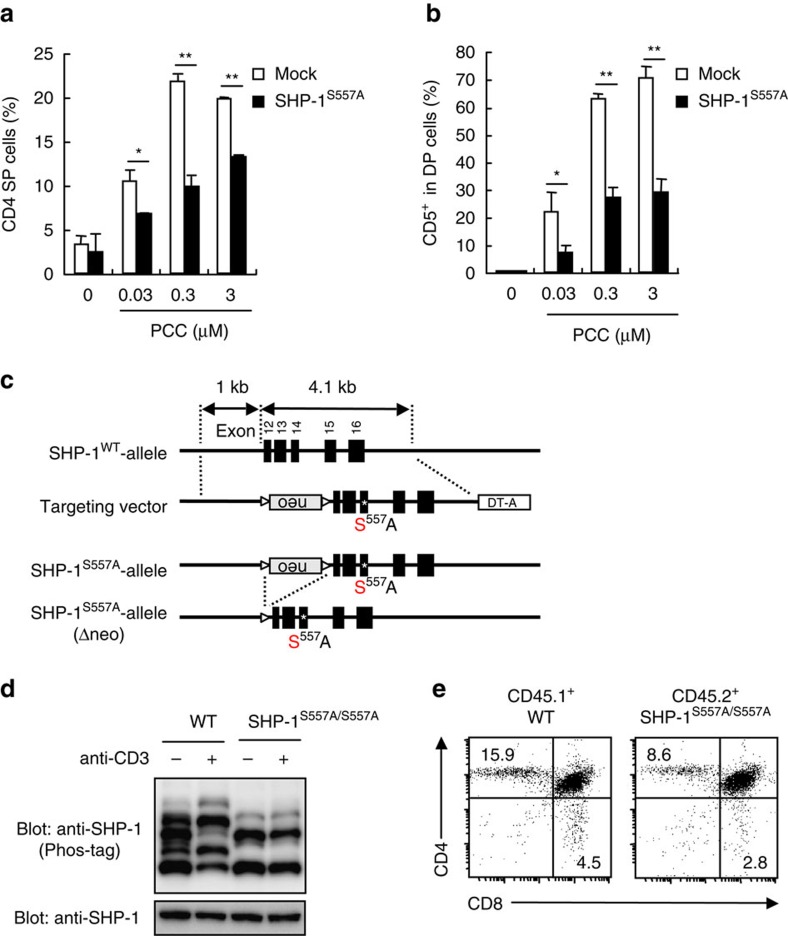
Phosphorylation of SHP-1 Ser^557^ is critical for T cell development. (**a**,**b**) DPK cells expressing vector alone (Mock) or mutant SHP-1 (SHP-1^S557A^) were stimulated with the indicated concentrations of PCC peptides presented on DCEK cells expressing I–E^k^. The percentages of CD4 SP thymocytes (**a**) and CD5^+^ cells in DP thymocytes (**b**) at day 3 were analysed by flow cytometry. *P<0.05, **P<0.01. (**c**) Targeting construct for establishment of SHP-1^S557A^ knock-in mice. (**d**) Phosphorylation and expression level of SHP-1 in unstimulated- and TCR-stimulated-WT and SHP-1^S557A/S557A^ thymocytes were examined by Phos-tag immunoblot analysis (upper panel) and Western blot analysis (lower panel). (**e**) Analysis of CD4 SP cell generation in thymocytes expressing phosphorylation-defective SHP-1^S557A^. CD3ɛ^Δ5/Δ5^ mice that were transferred with mixed BM cells from CD45.1^+^ WT and CD45.2^+^ SHP-1^S557A/S557A^ mice were analysed for CD4 and CD8 expression on thymocytes by flow cytometry 4 weeks after BMT. Data are presented as mean±s.d. of triplicate assays (**a**,**b**) and representative of three (**a**,**b**), two (**d**) or five (**e**) independent experiments. Unpaired two-tailed Student's *t* test is used to calculate *P* values.

To further examine the role of the SHP-1 phosphorylation in T cell development, we turned to the DPK cell line, a pigeon cytochrome *c* (PCC)-specific DP cell line that retains the potential to undergo differentiation *in vitro*^38^. Stimulation of DPK cells with cognate peptide on I–E^k^ resulted in downregulation of CD8 expression and differentiation into CD4 SP cells as previously reported^38^ (Fig. 10a, Mock). Forced expression of a phosphorylation-defective mutant of SHP-1 (SHP-1^S557A^) reduced the generation of CD4 SP cells (Fig. 10a). Likewise, SHP-1^S557A^ also inhibited CD5 upregulation induced by peptide stimulation (Fig. 10b). These results suggest that SHP-1^S557A^ can suppress TCR signalling in a dominant negative manner and that phosphorylation of Ser^557^ in SHP-1 promotes TCR signalling.

We finally examined the *in vivo* relevance of the phosphorylation of Ser^557^ in SHP-1 by establishing knock-in mice expressing mutant SHP-1 (SHP-1^S557A/S557A^ mice) (Fig. 10c). Thymocytes from SHP-1^S557A/S557A^ mice expressed comparable amount of SHP-1 to that of WT, whereas TCR-induced phosphorylation was impaired (Fig. 10d), indicating that Ser^557^ is a major phosphorylation site on TCR stimulation. To address the role of this site, we evaluated the ability of SHP-1^S557A/S557A^ thymocytes to develop into SP cells in the context of competition with WT. CD3ɛ-deficient (CD3ɛ^Δ5/Δ5^) mice lacking T cells were transferred with mixed BM cells from CD45.1^+^ WT and CD45.2^+^ SHP-1^S557A/S557A^ mice and analysed after T cell reconstitution. SHP-1^S557A/S557A^ thymocytes showed impaired development into CD4 SP population compared with WT thymocytes (Fig. 10e), indicating that Ser^557^ is required for the optimal generation of CD4 SP thymocytes. Collectively, these results suggest that the phosphorylation of Ser^557^ in SHP-1 can positively regulate TCR signalling in DP thymocytes to promote T cell development.

## Discussion

In this study, we established T cell-specific PKD-deficient mice to characterize the role of PKD in T cell development. PKD appears to control the generation of CD4 SP thymocytes through promoting (i) TCR signalling during positive selection, and (ii) CD4 lineage specification. Since the decreased number of CD4^+^CD8^int^ cells in PKD2/3^ΔT^ mice was restored by a Bcl-2 transgene (Fig. 3h), an important function of PKD during positive selection may be promoting survival that allows DP cells to differentiate into the CD4^+^CD8^int^ stage. On the other hand, defective CD4 lineage specification on loss of PKD is likely due to the incomplete induction of ThPOK during positive selection, as CD4/CD8 ratio was restored by ThPOK transgene, but not by Bcl-2. Optimal ThPOK induction requires ‘duration' of TCR signalling during positive selection^39^. In contrast, the expression of Runx3, a transcription factor essential for CD8 lineage specification, is controlled by TCR-induced immediate upregulation of IL-7R and subsequent IL-7 signalling^40^. As Runx3 induction occurred normally in PKD-deficient mice (Fig. 6b), PKD is apparently dispensable for the transient TCR signalling required for Runx3 induction. In this respect, we speculate that another critical role of PKD may be to maintain the ‘duration' of TCR/co-receptor signalling after sensing a weak-affinity ligand. Consistent with this idea, PKD was efficiently activated by stimulation with weak-affinity peptide variants in preselection DP thymocytes (Supplementary Fig. 5).

PKD2 is abundantly expressed in thymocytes, while the amount of PKD3 is considerably lower. Nevertheless, the strikingly different phenotype of PKD2/3^ΔT^ versus PKD2^ΔT^ mice demonstrates that a limited amount of PKD3 is sufficient to support SP thymocyte generation. The redundant functions of PKD2 and PKD3 in T cell development suggest that their substrate specificities are likely to overlap. Indeed, all substrates tested in this study were phosphorylated by both PKD2 and PKD3 with similar efficiencies (Fig. 8d). The PKD consensus phosphorylation motif has been reported to be L/V/IxRxxS/T^37^. The new phosphorylation sites that we have identified herein are similar but not identical to this motif (Fig. 8f). This finding underscores the important contribution of unbiased phospho-proteomics^33^,^41^, in addition to motif-based approaches^42^, in identifying novel kinase substrates.

It has been suggested that SHP-1 dephosphorylates several key molecules involved in TCR signalling mainly through interactions with ITIM-containing transmembrane proteins^17^. Recently, Themis, a molecule critical for positive selection, was reported to recruit SHP-1 to antagonize TCR signalling, thereby preventing the apoptosis of DP cells that encountered weak agonists during positive selection^43^, although some debate exists^44^. Given that loss of Themis or PKD cause similar defects in T cell development, one might speculate that PKD also antagonizes TCR signalling to ensure positive selection. However, this possibility seems less likely, as the expression levels of CD5, CD69 (Fig. 5f) and Nur77 (Supplementary Fig. 6), which represent the summation of TCR signalling intensity^45^,^46^, were reduced in PKD-deficient cells. This finding is consistent with the fact that active PKD induces upregulation of CD5 in thymocytes^15^. In addition, a ‘selection shift' from negative selection to positive selection has been reported in mice with defective TCR signalling, such as ZAP-70-mutated mice or CD3ζ^−/−^ mice^47^,^48^, similar to what we observe herein for PKD2/3^ΔT^ mice (Fig. 4e,f). In contrast, in Themis^−/−^ mice, proximal TCR signalling is enhanced rather than attenuated^43^. Therefore, although PKD and Themis can both act on SHP-1, they appear to regulate TCR signalling in opposite directions. The generation of Themis^−/−^ × PKD2/3^ΔT^ triple-deficient mice will clarify this issue.

What is the molecular basis for the positive signalling resulting from the phosphorylation of Ser^557^ in SHP-1? There are at least three possibilities: First, the phosphorylation by PKD may constrain the inhibitory function, such as phosphatase activity, of SHP-1. Alternatively, the phosphorylation might alter the proper cellular localization of SHP-1 away from the area where efficient inhibition of TCR signalling takes place. Indeed, a 6-mer motif including Ser^557^ in SHP-1 is reported to regulate its subcellular localization^49^. Second, we cannot rule out the possibility that serine-phosphorylated SHP-1 dephosphorylates proteins containing tyrosines that on phosphorylation, negatively regulate TCR signalling. For example, SHP-1 dephosphorylates the C-terminal regulatory tyrosine of Src family PTKs in osteoblasts to activate their kinase activities^50^. Third, SHP-1 may function as an adaptor rather than a phosphatase through its C-terminal phospho-Ser^557^, and may recruit positive regulators into the vicinity of the TCR. In every case, it would be extremely intriguing to identify the protein(s) that selectively bind to Ser^557^-phosphrylated SHP-1. Johnson *et al.*^51^ recently reported that T cell-specific SHP-1 deletion using *Cd4*-Cre Tg mice had no obvious effect on T cell development. This may be explained by the loss of both positive and negative regulation via SHP-1 in thymocytes lacking SHP-1 protein.

The observation that the defects found in SHP-1^S557A/S557A^ mice are milder than that of PKD2/3^ΔT^ mice could be explained by a possible contribution of other PKD substrates, such as Gads and NCK1. Indeed, co-transfection of phosphorylation-defective forms of three other PKD substrates (Gads, NCK1 and pro-IL-16) together with SHP-1^S557A^ resulted in more severe impairment in CD5 upregulation (Supplementary Fig. 7). Thus, we cannot exclude the possibility that PKD might control T cell development by modulating multiple TCR signalling pathways through various substrates in addition to SHP-1.

Histone deacetylase 7 (HDAC7) is also reported to be a substrate for PKD^52^ and we confirmed this in *in vitro* kinase assay (Fig. 8d), whereas we could not detect PKD-dependent phosphorylation of HDAC7 in thymocytes by 2D-DIGE (Fig. 8a,b). HDAC7-deficient mice show poor generation of SP thymocytes^53^. However, the phenotype of PKD-deficient mice is unlikely to be attributed to HDAC7, as loss of HDAC7 results in unique phenotypes, such as limited usage of TCR Jα segments, which are not observed in PKD2/3^ΔT^ mice. In addition, in HDAC7-deficient mice, the introduction of TCR transgenes (OT-I or OT-II) reduced rather than exacerbated the defect in thymocyte development^53^, which contrasts with the phenotypes seen in PKD-deficient mice.

Currently, the pathway upstream of PKD during thymic selection is not clearly understood. PKD is activated by binding to DAG and, more importantly, by phosphorylation of serine residues in the activation loop (Ser^707/711^ in PKD2 and Ser^731/735^ in PKD3)^3^. In T cells, classical as well as novel PKCs, including PKCα, δ, ɛ, η and θ, are implicated in the phosphorylation of these residues^14^. Among them, PKCθ and PKCη are abundantly expressed in DP thymocytes, and recently, PKCθ^−/−^PKCη^−/−^ double-deficient mice were demonstrated to have a decreased number of SP thymocytes compared with single-deficient mice^54^. In this regard, one can speculate that PKDθ and PKCη act as upstream regulators of PKD after TCR engagement in thymocytes. This hypothesis warrants further investigation.

In conclusion, we propose that the PKD represents one of the molecular switches that converts weak TCR engagement to positive selection and CD4 lineage specification. The critical roles of PTKs and PTPs in thymic selection have been clearly established. The present study brings to light the additional importance of serine/threonine kinases as key modulators of TCR signalling cascade regulated by tyrosine phosphorylation during thymic selection.

## Methods

### Mice

PKD2-floxed and PKD3-floxed mice were generated by homologous recombination-mediated gene targeting in embryonic stem (ES) cells of C57BL/6 and 129 genetic background, respectively. PKD3-floxed mice were backcrossed to C57BL/6 mice and floxed *Neo* cassette of each allele was deleted by crossing with CAG-Cre Tg mice. Bcl-2 Tg mice^55^ were provided by J. Domen (The Children's Mercy Hospital, Kansas City, MO) by the courtesy of K. Ikuta (Kyoto University, Kyoto, Japan). CD3ɛ^Δ5/Δ5^ mice^56^ were provided by B. Malissen (Aix Marseille Université UM2, Marseille, France). Truncated PKD2 (a.a. 1-381) Tg mice were established under *Lck* proximal promoter^57^. Tg mice expressing ThPOK were established under *Cd4* enhancer and promoter^58^. For the generation of SHP-1^S557A/S557A^ mice, C57BL/6-derived ES cells were co-transfected with a circular form of targeting vector and two Cas9D10A and sgRNA expression vectors (pX335, Addgene) to produce nicks in homology arms and genomic DNA for efficient homologous recombination (gRNAs for CRISPR were GTGGTGGA GTGGGAGAAGCCC (pX335-SHP-short arm) and AGCTATGATATGGCTTCACA (pX335-SHP-long arm)). A floxed *Neo* cassette was deleted by crossing with CAG-Cre Tg mice. Male and female mice of 7–12 weeks of age with C57BL/6 background were used for all experiments unless otherwise specified. For *in vivo* negative selection model, C57BL/6 (H-2^b^) mice and B10.D2 (H-2^d^) mice were obtained from Kyudo Co. Ltd. and Sankyo Labo Service Co. Inc., respectively. All mice were maintained in filter-air, laminar-flow enclosures and given standard laboratory food and water *ad libitum*. Animal protocols were approved by the committee of Ethics on Animal Experiment, Faculty of Medical Sciences, Kyushu University.

### Cells and reagents

DP thymocyte cell line, DPK cells^59^ were obtained from J. G. Kaye (The Scripps Research Institute, La Jolla, CA, USA) and maintained in RPMI1640 medium (SIGMA) supplemented with 10% fetal bovine serum (FBS) (SIGMA), 100 U ml^−1^ penicillin, 10 μg ml^−1^ streptomycin, 50 μM 2-mercaptoehthanol, 0.1 mM non-essential amino acids and 1 mM sodium pyruvate. Murine fibroblast cell line DCEK transfected with I–E^k^ was kindly provided by K. Yasutomo (Tokushima University, Tokushima, Japan) and maintained in DMEM medium (SIGMA) supplemented with 10% FBS, 100 U ml^−1^ penicillin, 10 μg ml^−1^ streptomycin and 50 μM 2-mercaptoehthanol. To use DCEK cells as APC in *in vitro* DPK differentiation assay, cells were treated with 50 μg ml^−1^ mitomycin C for 30 min, washed with DMEM medium three times and plated on 96-well plates at 1 × 10^5^ cells per well. After 24 h, PCC_88–104_ peptides were added, and 2 h later, 5 × 10^4^ DPK cells were plated. The mixed culture was incubated for 3 days and analysed by flow cytometry for the expression of CD4, CD8 and CD5. Anti-PKD1/2/3 (pan) and anti-phospho-PKD1/2/3 (pan) were raised by immunization of rabbits with peptides, CIIGEKSFRRSVVGTP and CIIGEKpSFRRpSVVGTP, respectively, which are common sequence of PKD1, PKD2 and PKD3. Specificity of these Abs was validated by Western blotting of lysates from HEK293 cells that were transfected with PKD1, PKD2 and PKD3 or WT PKD2, PKD2^S707A^, PKD2^S711A^ and PKD2^S707/711A^ (Fig. 1a). Anti-PKDs Ab and anti-pPKDs Ab were used at 10 and 2 μg ml^−1^, respectively. Anti-pSHP-1 (Ser^557^) was raised with a peptide, CASRTSpSKHKEE and used at 2 μg ml^−1^. Anti-CD3ɛ (cat#553058, 145-2C11, 10 μg ml^−1^), anti-CD4 (cat#553051, RM4-5, 1:100), anti-CD8 (cat#553033, 53-6.7, 1:100), anti-CD69 (cat#553236, H1.2F3, 1:100), anti-Vα2 (cat#553288, B20.1, 1:80), anti-mouse TCR Vβ screening panel (cat#557004, 1:2) and anti-pCD247 (Y^142^) (pCD3ζ) (cat#558486, 1:50) were purchased from BD Biosciences. Anti-CD5 (cat#100606, 53-7.3, 1:50), anti-TCRβ (cat#109206, H57, 1:20) and anti-CD4 (cat#100540, RM4-5, 1:100) were from BioLegend. Anti-H–Y TCR (cat#11-9930-82, T3.70, 1:100), anti-TCRβ (cat#16-5961, H57) and anti-CD2 (cat#16-0021, RM2-5) were purchased from eBioscience. Annexin V-FITC kit (cat#4700, 1:20) was from MBL. For intracellular staining, anti-pErk1/2 (anti-ACTIVE MAPK) (cat#V8031, 1:40) was obtained from Promega and anti-pZAP70 (cat#2717, 1:50), anti-pp38 (cat#4511, 1:50) and anti-pJNK (cat#9255, 1:25) were from Cell Signaling Technology. For Western blotting, anti-pErk1/2 (cat#4379, 1:2,000), anti-Erk1/2 (cat#9102, 1:1,000), anti-NCK1 (cat#2319, 1:000) and anti-β-actin (cat#4970, 1:1,000) were obtained from Cell Signaling Technology. Anti-SHP-1 (cat#sc-287, 1:200) was from Santa Cruz Biotechnology. Anti-Gads (cat#06-983, 2.5 μg ml^−1^) was from Upstate Biotechnology. Goat anti-hamster IgG (cat#55397) was from MP Biomedicals. OT-I TCR-specific OVA_257–264_ peptide (SIINFEKL) and OT-II TCR-specific OVA_323–339_ peptide (ISQAVHAAHAEINEAGR) were from ABGENT. OVA_323–339_ variants, F9 and R9, were from ABGENT and MBL, respectively. OVA_257–264_ peptide variants, T4 (SIITFEKL) and G4 (SIIGFEKL), were purchased from AnaSpec.

### Immunoblotting

Preselection DP thymocytes were obtained from TAP-deficient mice transferred with OT-I Tg BM cells. For peptide stimulation, preselection DP thymocytes were added with 10 μM OVA peptides followed by centrifugation at 4 °C for packing, and stimulated at 37 °C. For CD3 cross-linking, cells were stained with 10 μg ml^−1^ anti-CD3 Ab at 4 °C for 30 min, washed twice with cold medium, and then cross-linked by 100 μg ml^−1^ goat anti-hamster IgG at 37 °C. After stimulation, cells were rapidly transferred on ice and added with cold HEPES buffered saline for washing. Then, cells were lysed in lysis buffer containing 1% Nonidet P-40, 1 mM PMSF, protease inhibitor cocktail, 10 mM NaF and 1 mM Na_3_VO_4_ and the lysates were mixed with sample buffer and applied to western blot analysis^60^. For Phos-tag immunoblotting, 50 U ml^−1^ benzonase (Novagen) was also added in RIPA buffer. Images have been cropped for presentation. Full size images are presented in Supplementary Figs 8–12.

### Quantitative RT-PCR

For RT-PCR, following primers were used: PKD1 (F: 5′-ATAACACTCTTCCAAAATGACACAGG-3′, R: 5′-TCTCCCACATAATACACTACATTCGC-3′), PKD2 (F: 5′-GATGAGTTGGAGGATTCTGGTGTC-3′, R: 5′-AGGGTGGTGCTGGATTTCCG-3′), PKD3 (F: 5′-AAGAGACATTATTGGAGACTTGACAG-3′, R: 5′-AGTGTGGGTTACTGCCTTGTG-3′), Bcl-2 (F: 5′-TCCTTCCAGCCTGAGAGCAAC-3′, R: 5′-CACGACGGTAGCGACGAGA-3′), ThPOK (F: 5′-GGAGCAGAGCCCCAAGCC-3′, R: 5′-GATTCCAATCAGGTCATCCTCGG-3′), Runx3 (F: 5′-AGCACCACGAGCCACTTCAG-3′, R: 5′-TAGGGAAGGAGCGGTCAAACTG-3′), Runx3d (F: 5′-AGCACGTCCACCATCGAG-3′, R: 5′-TGCGACATGGCTTCCAACAG-3′), Nur77 (F: 5′-CCACCTCTCCGAACCGTGACA-3′, R: 5′-GAGAAGATTGGTAGGGGAGGC-3′).

### Molecular indexing assay

RNA extracted from DP thymocytes of WT mice was reverse-transcribed with oligo(dT) primers containing molecular indexing labels. ds cDNA was amplified with gene-specific primers and fluorescent dye-labelled universal primers. The number of labels in amplified ds cDNA was detected by microarray hybridization with Pixel System (Cellular Research).

### Cell survival assay

Thymocytes were cultured in medium at 37 °C with 5% CO_2_. After incubation for different times, cells were stained with APC-conjugated anti-CD4, PE-conjugated anti-CD8, FITC-conjugated Annexin V at the concentration described in the ‘Cells and reagents' section and propidium iodide at room temperature for 15 min, and immediately analysed by flow cytometry.

### *In vivo* negative selection model

C57BL/6 (H-2^b^) mice were backcrossed onto B10.D2 (H-2^d^) mice and analysed for the expression of Vβ5, Vβ6 and Vβ11. Thymocytes expressing Vβ5 or Vβ11 are deleted by binding of Mtv-8 and Mtv-9 superantigens only when I–E is also expressed. Thus, deletion occurs on H-2^b/d^ but not H-2^b/b^ backgrounds. Thymocytes expressing Vβ6 are not deleted by these superantigens.

### Two-step differentiation assay

*In vitro* differentiation of CD4^+^CD8^int^ thymocytes was performed using purified DP thymocyets^26^. Thymocytes in RPMI1640 medium supplemented with 10% (v/v) charcoal- and dextran-treated FBS were plated on 96-well plates at 5 × 10^5^ cells per well and stimulated for 20 h in wells coated with 5 μg ml^−1^ anti-TCRβ and 10 μg ml^−1^ anti-CD2. Cells were washed and immediately analysed for CD4 and CD8 expressions by flow cytometry (stimulatory culture) or transferred to a new plate, washed extensively, and cultured for another 20 h in same medium, followed by the analysis for the expression of CD4 and CD8 by flow cytometry (recovery culture).

### Co-receptor downregulation assay (Dulling assay)

Co-receptor downregulation was examined using OT-II thymocytes or preselection OT-I thymocytes^27^. Overall, 1 × 10^5^ thioglycollate-induced peritoneal cells were used as APC and pulsed with peptides before the addition of thymocytes (1.5 × 10^5^–5.0 × 10^5^) for stimulation of OT-II thymocytes. To stimulate preselection OT-I thymocytes, peptides were loaded on MHC class I expressed on thymocytes themselves. The expression of CD4 and CD8 was analysed by flow cytometry after 16–20 h.

### BM chimera for quantification of redirection

For the analysis of CD8-redirection, BM cells from WT or PKD2/3^ΔT^ mice were suspended in phosphate-buffered saline (PBS) and 1 × 10^7^ cells were injected into 8 Gy-irradiated TAP^−/−^ mice. Then, 6 weeks after BMT, mice were dissected and thymocytes were analysed. For mixed BM transfer, CD3ɛ^Δ5/Δ5^ mice were transferred with 5 × 10^6^ each of CD45.1^+^ WT and CD45.2^+^ SHP-1^S557A/S557A^ BM cells and analysed 4 weeks after BMT.

### Ca^2+^ mobilization

Thymocytes were freshly isolated and loaded with 1.2 μM Fluo-4 (Molecular Probes) in phenol red-free RPMI1640 containing 5% FBS for 30 min at 30 °C. After washing, cells were stained with anti-CD3, phycoerythrin-conjugated anti-CD8 and PerCP-Cy5.5 -conjugated anti-CD4 at the concentration described in the ‘Cells and reagents' section for 30 min on ice. Cells were washed, suspended in PBS containing 5% FBS, prewarmed to 37 °C before analysis and were kept at 37 °C during event collection on FACSCalibur (Becton-Dickinson). For cell stimulation, various concentrations of goat anti-hamster IgG were added to crosslink anti-CD3 Abs. Maximum Ca^2+^ influx was obtained by the addition of 10 μM ionomycin (Calbiochem). Mean fluorescence ratio was calculated by FlowJo software (Tree Star).

### Intracellular staining

For intracellular staining of phosphorylated proteins, thymocytes were stained with anti-CD3, anti-CD4 and anti-CD8 at the concentration described in the ‘Cells and reagents' section for 30 min on ice and cross-linked by goat anti-hamster IgG as described above. Cells were washed, fixed and permeabilized using cytofix/cytoperm solution (BD Biosciences) for pCD3ζ, pErk, pp38 and pJNK staining or fixation/permeabilization buffer (eBioscience) for pZAP70 staining. Data were analysed by FACSCalibur flow cytometer and CellQuest software (BD Biosciences).

### 2D-DIGE

Thymocytes were freshly isolated and cultured for 16 h to upregulate TCR. Cells were then incubated with 10 μg ml^−1^ anti-CD3 mAb for 30 min on ice and stimulated by cross-linking by 100 μg ml^−1^ goat anti-hamster IgG Ab for 2 min at 37 °C. The unstimulated WT, stimulated WT and stimulated PKD2/3^ΔT^ cells were lysed and phosphoproteins were enriched using PhosphoProtein Purification Kit (Qiagen). The interfering non-protein materials were subsequently removed by 2-D Clean Up Kit (GE Healthcare). Overall, 30 μg of the resultant phosphoproteins were minimally labelled with 400 pmol Cy2, Cy3 or Cy5 fluorescent dye (CyDye DIGE Fluors, GE Healthcare) for 30 min on ice. After quenching the labelling reaction with 10 nmol lysine, differentially labelled samples were mixed and subjected to first-dimension isoelectric focusing on immobilized pH gradient strips (24 cm; pH4–7) using an Ettan IPGphor II system. The second-dimensional SDS–PAGE was carried out on 10% acrylamide gel. The Cy2, Cy3 and Cy5 signals were individually acquired with a Typhoon 9400 scanner (GE Healthcare).

### Phos-tag western blotting

Lysates from thymocytes were subjected to PAGE clean up kit (Nacalai tesque) before phosphate-affinity SDS–PAGE is performed using 6–7.5% acrylamide gels containing 25 or 50 μM Phos-tag acrylamide (Wako chemicals) and 50 or 100 μM MnCl_2_. After electrophoresis, gels were washed in Trans-Blot Turbo transfer buffer (Bio-Rad) added with 1 mM EDTA for 15 min and then incubated in the buffer without EDTA for 15 min. Proteins were transferred to polyvinylidene difluoride (PVDF) membranes using Trans-Blot Turbo Transfer System (Bio-Rad) with the program for HIGH molecular weight proteins (1.3A, 25 V, 10 min) and analysed by immunoblotting. For λ protein phosphatase (λ PP) treatment, lysates were treated with λ PP for 30 min at 30 °C before purification using PAGE clean up kit.

### Preparation of GST fusion proteins

SHP-1, Gads and NCK1 cDNAs were cloned into pGEX-6P-1 vector (GE Healthcare). For the mutant GST fusion proteins, Ser^557^ and Ser^591^ of SHP-1, Ser^186^ of Gads and Ser^85^ of NCK1 were changed to alanine by site-directed mutagenesis. The vectors were transformed into *E. coli* BL21 and GST fusion proteins were purified using glutathione sepharose (GE Healthcare).

### *In vitro* kinase assay

For *in vitro* kinase assay using [γ-^32^P]ATP, 2 μg GST-SHP-1, GST-Gads and GST-NCK1 were incubated with 50 ng active PKD2 or PKD3 (Carna Bioscience) and 100 μM [γ-^32^P]ATP (5 μCi) in 20 μl kinase buffer (5 mM MgCl_2_ and 20 mM Tris–HCl, pH7.5) for 30 min at 30 °C. The reaction was stopped by addition of sample buffer followed by heating at 95 °C for 5 min. Half of the sample was subjected to 5–20% gradient SDS–PAGE and CBB staining. Phosphorylated proteins were visualized by autoradiography.

### Identification of proteins and peptides by LC–MS/MS analysis

The 2D-DIGE gel was silver-stained (Thermo Scientific) and protein spots were excised and digested with trypsin (Promega). The obtained peptides were analysed by a capillary liquid chromatography system (Waters/Micromass) connected to a Q-TOF Ultima mass spectrometer (Waters/Micromass). Raw data were acquired and processed using MassLynx version 4.0. (Waters/Micromass) to generate a peak list file for MS/MS ion search. The peak list files were searched against the NCBI non-redundant protein database restricted *Mus musculus* using the MS/MS ion search on Mascot search engine (Matrix Science). To identify *in vitro* phosphorylation sites, GST fusion proteins phosphorylated by active PKD2 or PKD3 were subjected to SDS–PAGE. The gel was stained with Coomassie Brilliant Blue and protein bands were excised and digested with trypsin. The resultant peptides were analysed by an ADVANCE UHPLC (Michrom Bioresources) connected to an Orbitrap Velos Pro mass spectrometer (for GST-Gads and GST-NCK1) or by an EASY-nLC1000 UHPLC (Thermo Fisher Scientific) connected to an Orbitrap Fusion Tribrid mass spectrometer (for GST-SHP-1).

### Identification of phosphopeptides by LC–MS/MS analysis

The unstimulated WT, stimulated WT and stimulated PKD2/3^ΔT^ thymocytes were lysed in buffer containing 50 mM Tris–HCl, pH 8.0, 8 M urea, 1 mM DTT and PhosSTOP phosphatase inhibitor mixture (Roche). The lysates were sonicated and cleared by centrifugation at 20,000*g* at 4 °C for 15 min. Proteins (each 50 μg) were reduced with 5 mM DTT for 30 min and alkylated with 27.5 mM iodoacetamide for 30 min in the dark. The urea concentration was then reduced to 1 M before addition of 0.5 μg trypsin. After 4 h at 37 °C, the digestion was quenched by acidification with 0.5% TFA. The digests were desalted using InertSep RP-1 (GL Sciences), concentrated and adjusted to binding conditions for TiO_2_-based phosphopeptide enrichment (80% acetonitrile, 0.5% TFA and 300 mg ml^−1^ lactic acid). Phosphopeptides were enriched using 50 mg of Titansphere Phos-TiO column (GL Sciences) in accordance with the manufacturer's instructions. Peptides were eluted with 200 μl of 5% ammonia and then with 200 μl of 5% pyrrolidine. The collected eluates were acidified with TFA and desalted using GL-Tip SDB (GL Sciences). LC–MS/MS analysis of enriched phosphopeptides was performed on an EASY-nLC 1200 UHPLC (Thermo Fisher Scientific) connected to a Q Exactive Plus mass spectrometer (Thermo Fisher Scientific). The peptides were separated on an EASY-Spray column (15 cm × 75 μm, PepMap C18, 3 μm) with a linear gradient from 4 to 28% ACN in 0–100 min followed by increase to 80% ACN in 100–110 min. The mass spectrometer was operated in a data-dependent acquisition mode with a top 10 MS/MS method. Raw data were converted to the mgf files using Proteome Discoverer version 2.0 (Thermo Fisher Scientific), which were then searched against the SwissProt database restricted to *M. musculus* using the MS/MS ion search on Mascot search engine. The false discovery rate was set to be <0.01. To generate extracted ion chromatograms, the raw data were processed using Xcalibur software (Thermo Fisher Scientific).

### Data availability

The mass spectrometry proteomics have been deposited in the jPOST repository (https://repository.jpostdb.org) under the accession codes JPST000031, JPST000032 and JPST000033. All other data supporting the findings of this study are available within the article and Supplementary Information, and can also be obtained from the corresponding author upon request.

## Additional information

**How to cite this article:** Ishikawa, E. *et al.* Protein kinase D regulates positive selection of CD4^+^ thymocytes through phosphorylation of SHP-1. *Nat. Commun.* 7:12756 doi: 10.1038/ncomms12756 (2016).

## Supplementary Material

Supplementary Figures Supplementary Figures 1-12

Supplementary Data 1 A complete list of proteins and peptides identified by LC-MS/MS analysis of six "red" spots in the 2D-DIGE gel. This table is related to Fig. 8a,b.

Supplementary Data 2 A complete list of peptides and phosphopeptides identified by LC-MS/MS analysis of in vitro phosphorylated GST fusion proteins. GST-fused SHP-1, Gads and NCK1 were incubated with buffer, PKD2 or PKD3 plus ATP. This table is related to Supplementary Fig. 3.

Supplementary Data 3 A complete list of TiO_2_-enriched phosphorylated peptides identified by LC-MS/MS analysis of tryptic digests of unstimulated WT, TCR-stimulated-WT and PKD2/3^ΔT^ thymocyte lysates. This table is related to Fig. 9b,c and Supplementary Fig. 4.
