# Supplementary material for: Protein kinase D regulates positive selection of CD4+ thymocytes through phosphorylation of SHP-1
Source: Nat Commun. 2016 Sep 27;7:12756. doi: 10.1038/ncomms12756 (PMC5052653; doi:10.1038/ncomms12756)
Supplement: Supplementary Figures — 1-12 [file ncomms12756-s1.pdf]

Supplementary Figure 1

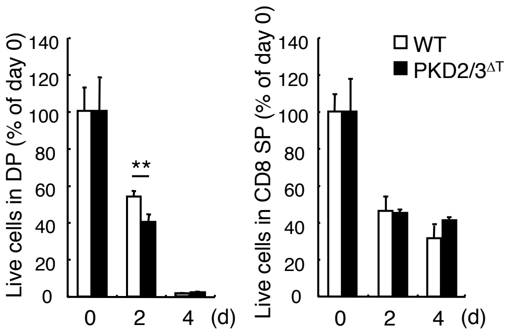

Supplementary Figure 1. *In vitro* survival of DP and CD8 SP thymocytes.
Total thymocytes from WT and PKD2/3$^{\Delta T}$ mice were cultured *in vitro* and the live cell number of DP or CD8 SP cells was analyzed by staining with Annexin V and propidium iodide after the indicated numbers of days. **, $p < 0.01$. Data are presented as mean ± SD of triplicate assays and representative of four independent experiments. Unpaired two-tailed Student's t test is used to calculate p values.

Supplementary Figure 2

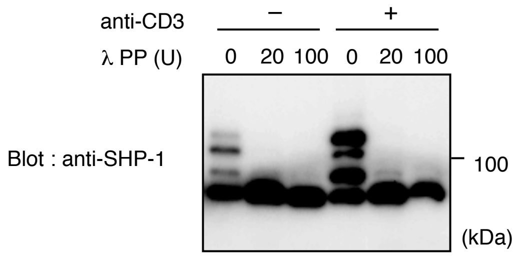

Supplementary Figure 2. Detection of phosphorylated SHP-1 as retarded bands by Phos-tag immunoblot analysis.

Lysates from unstimulated- or TCR-stimulated-thymocytes were treated with λ protein phosphatase (λ PP) and analyzed by Phos-tag immunoblotting using anti-SHP-1 Ab. Data are representative of two independent experiments.

Supplementary Figure 3

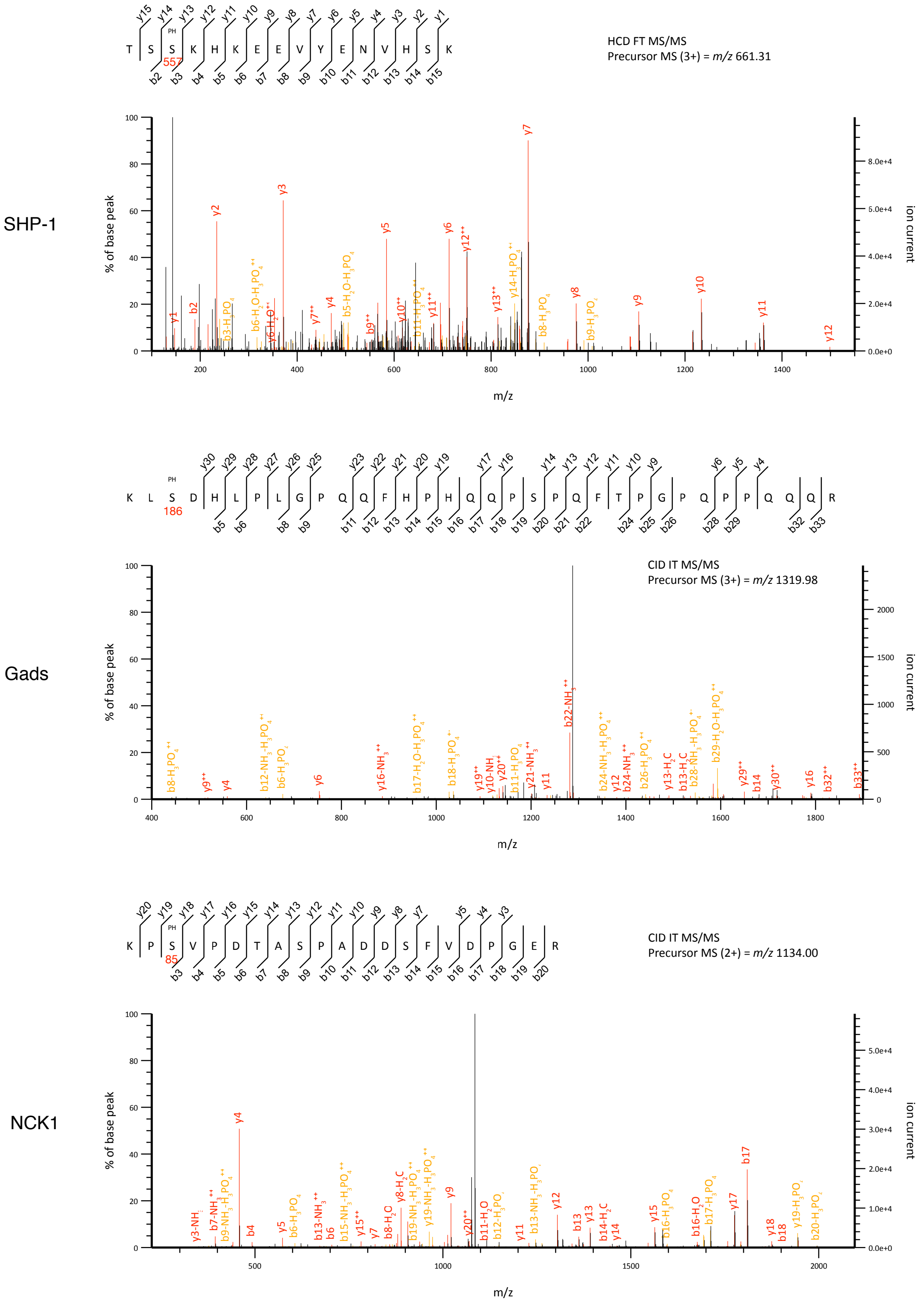

Supplementary Figure 3. LC-MS/MS analysis of GST-fused SHP-1, Gads and NCK1 phosphorylated by PKD2 *in vitro*. Annotated MS/MS spectra of the identified phosphopeptides are shown. Data are representative of two independent experiments.

Supplementary Figure 4

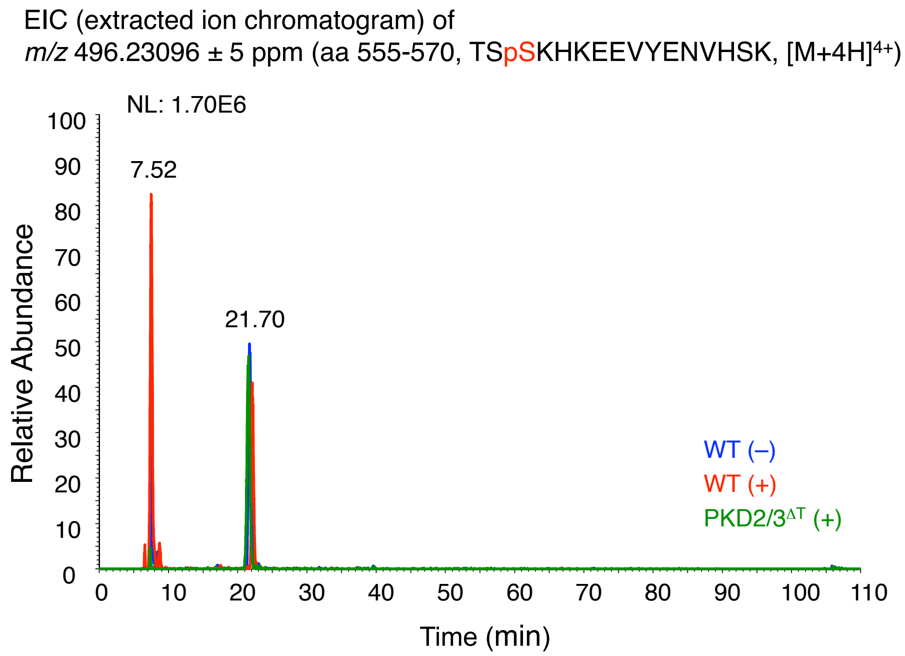

Supplementary Figure 4. LC-MS/MS analysis of endogenous SHP-1 phosphorylation in thymocytes. Tryptic phosphopeptides were enriched from unstimulated WT thymocytes and TCR-stimulated-WT and PKD2/3$^{\Delta T}$ thymocytes followed by LC-MS/MS analysis. The extracted ion chromatograms of *m/z* 496.23096 are shown over the entire LC run. Data are representative of two independent experiments.

Supplementary Figure 5

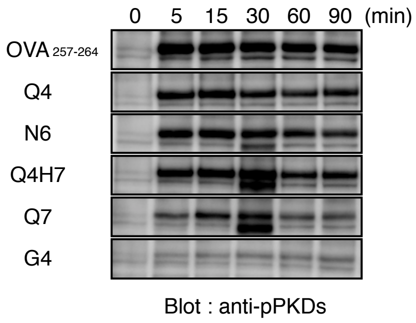

Supplementary Figure 5. Phosphorylation of PKD upon stimulation with OVA peptide variants.

Preselection OT-I DP thymocytes were stimulated with a variety of OVA peptides (10 μM) for the indicated times and phosphorylation of PKD was analyzed by anti-pPKDs. Data are representative of two independent experiments.

Supplementary Figure 6

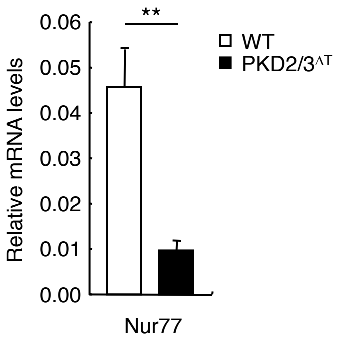

Supplementary Figure 6. Real time PCR analysis of Nur77 mRNA expression.
Total mRNA was extracted from CD4^{+}CD8^{int} thymocytes and analyzed for Nur77 expression by real time PCR. Results are presented as relative expression of β-actin. **, $p < 0.01$. Data are presented as mean ± SD of triplicate assays and representative of two independent experiments. Unpaired two-tailed Student's t test is used to calculate a p value.

Supplementary Figure 7

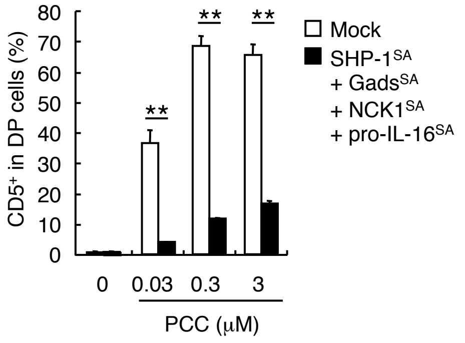

Supplementary Figure 7. CD5 expression in DPK cells expressing phosphorylation-defective forms of four PKD substrates.

DPK cells simultaneously expressing phosphorylation-defective mutants of SHP-1, Gads, NCK1 and pro-IL-16 were stimulated with the indicated concentrations of PCC peptides and the percentage of $CD5^+$ cells in DP thymocytes at day 3 was analyzed by flow cytometry. **, $p < 0.01$. Data are presented as mean ± SD of triplicate assays and representative of three independent experiments. Unpaired two-tailed Student's *t* test is used to calculate *p* values.

Supplementary Figure 8

Figure 1a

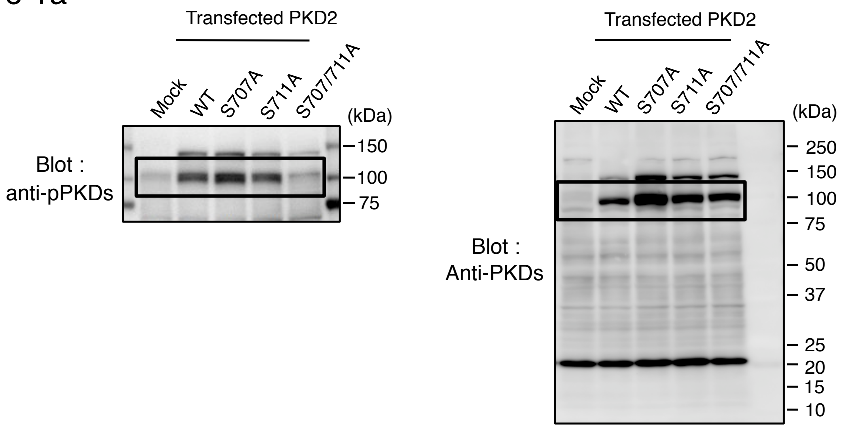

Figure 1b

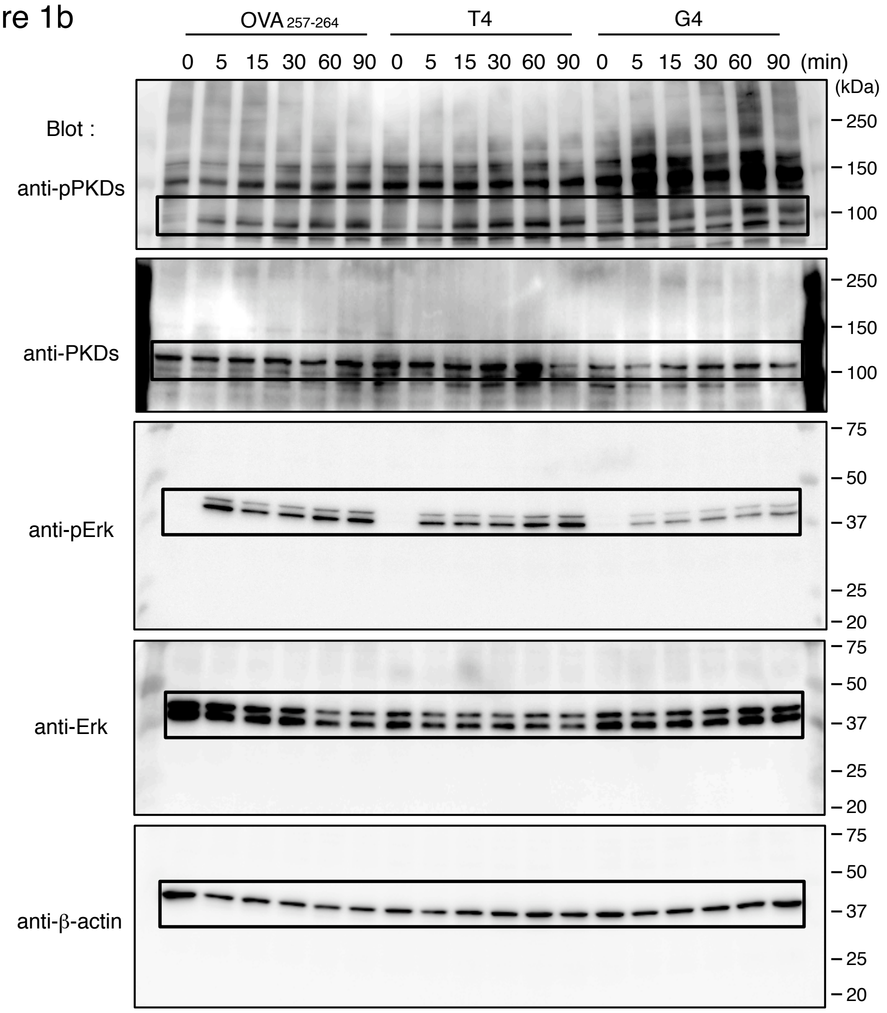

Supplementary Figure 8. Full size images of Immunoblot analyses in Figure 1a and 1b.

Supplementary Figure 9

Figure 2c

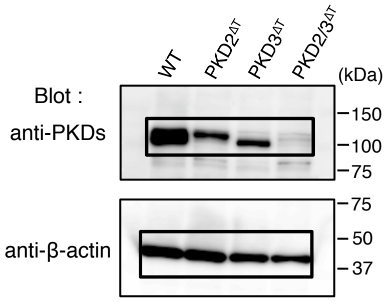

Figure 2d

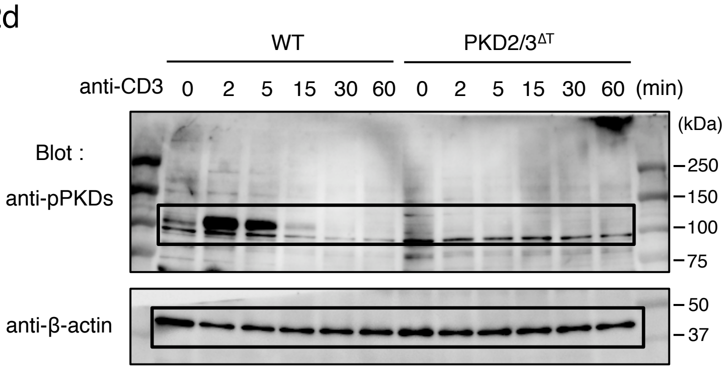

Supplementary Figure 9. Full size images of Immunoblot analyses in Figure 2c and 2d.

Supplementary Figure 10

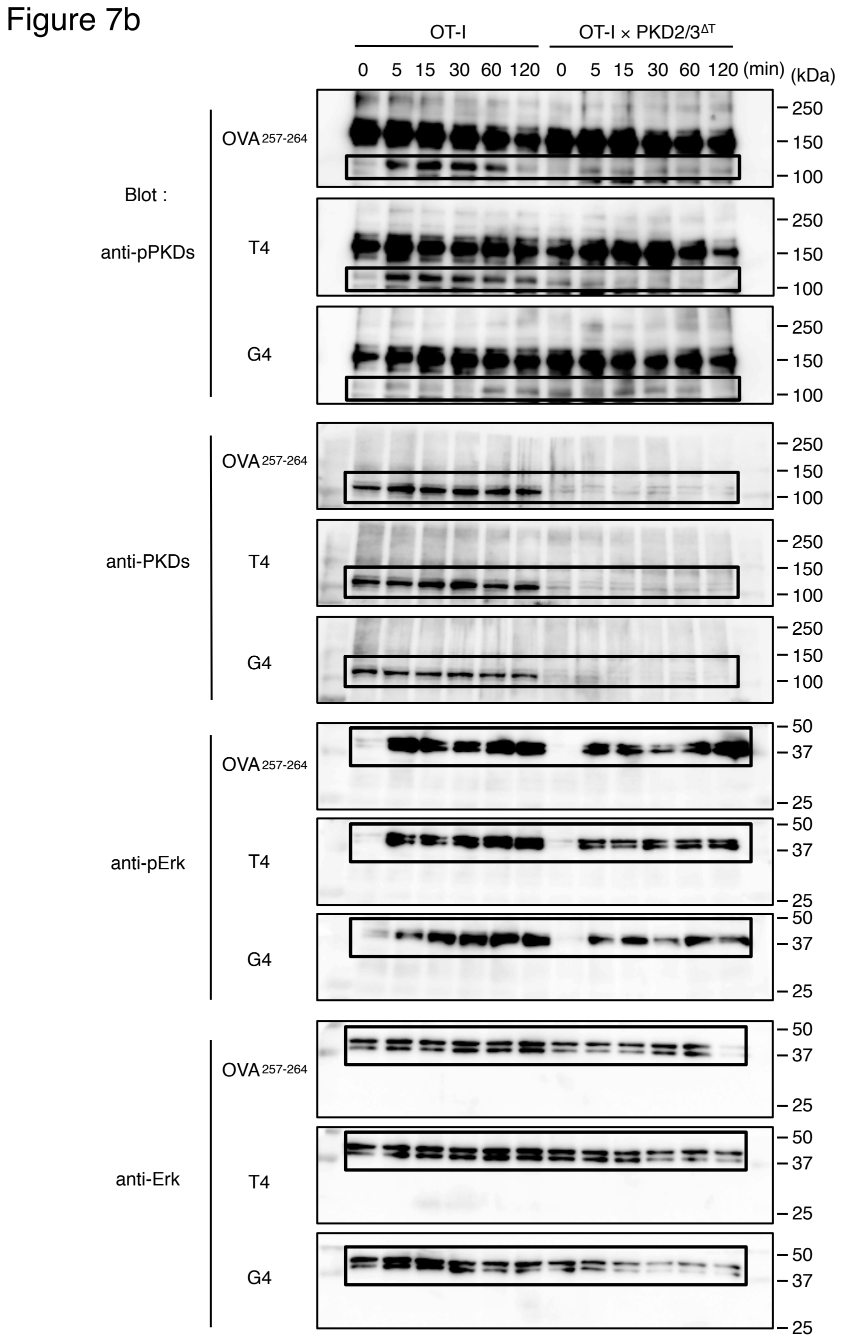

Supplementary Figure 10. Full size images of Immunoblot analyses in Figure 7b.

Supplementary Figure 11

Figure 8c

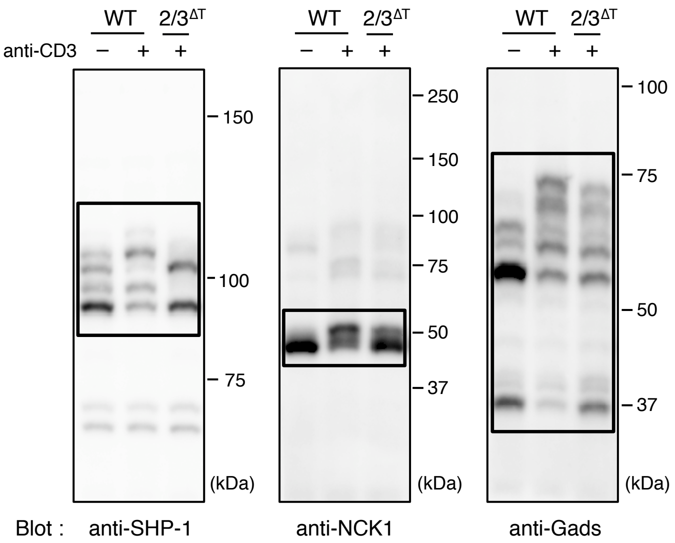

Supplementary Figure 2

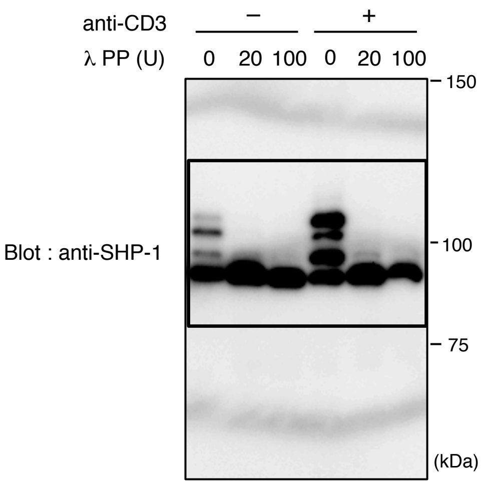

Supplementary Figure 11. Full size images of Immunoblot analyses in Figure 8c and Supplementary Figure 2.

Supplementary Figure 12

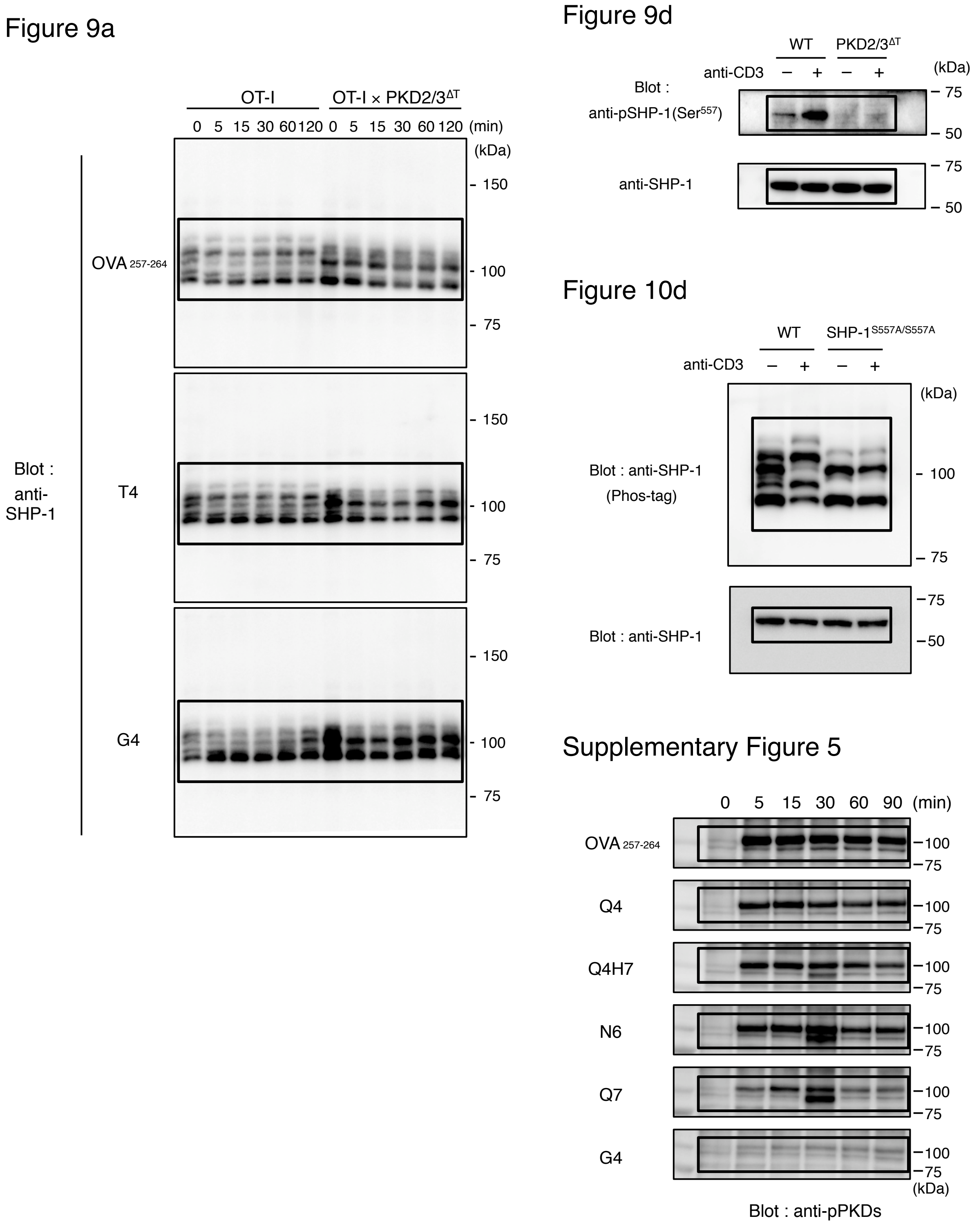

Supplementary Figure 12. Full size images of Immunoblot analyses in Figure 9a, 9d, 10d and Supplementary Figure 5.
